# Pediatric Complex Chronic Condition System Version 3

**DOI:** 10.1001/jamanetworkopen.2024.20579

**Published:** 2024-07-15

**Authors:** James A. Feinstein, Matt Hall, Amber Davidson, Chris Feudtner

**Affiliations:** 1 Adult and Child Center for Outcomes Research and Delivery Science, University of Colorado Anschutz Medical Campus, Aurora; 2 Children’s Hospital Colorado, Aurora; 3 Department of Pediatrics, University of Colorado Anschutz Medical Campus, Aurora; 4 Children’s Hospital Association, Lenexa, Kansas; 5 Division of General Pediatrics, Department of Pediatrics, Children’s Hospital of Philadelphia, Philadelphia, Pennsylvania

## Abstract

**Importance:**

Since implementation of the *International Statistical Classification of Diseases, Tenth Revision, Clinical Modification* (*ICD-10-CM*) in the US, thousands of new or related codes have been added to represent clinical conditions. The widely used pediatric complex chronic condition (CCC) system required a major update from version 2 (V2) to version 3 (V3) to capture the range of clinical conditions represented in the *ICD-10-CM*.

**Objective:**

To update the CCC V3 system, creating V3, with new, missing, or retired codes; to reconceptualize the system’s use of technology codes; and to compare CCC V3 with V2.

**Design, Setting, and Participants:**

This repeated cross-sectional study examined US hospitalization data from the Pediatric Health Information System (PHIS) and the Medicaid Merative MarketScan Research Databases from January 1, 2009, to December 31, 2019, for all patients aged 0 to 18 years. Data were analyzed from March 1, 2023, to April 1, 2024.

**Exposures:**

The CCCs were identified in both data sources using the CCC V2 and V3 systems.

**Main Outcomes and Measures:**

The (1) percentage of pediatric hospitalizations associated with a CCC, (2) numbers of CCC body-system categories per patient, and (3) explanatory power for hospital length of stay and in-hospital mortality were compared over time for V3 vs V2.

**Results:**

Among 7 186 019 hospitalizations within PHIS, 54.3% patients were male, the median age was 4 years (IQR, 1-11 years), and 51.2% were aged 0 to 4 years). The CCC V2 identified 2 878 476 (40.1%) patients as having any CCC compared with 2 753 412 (38.3%) identified by V3. In addition, V2 identified 100 065 (1.4%) patients with transplant status compared with 146 683 (2.0%) by V3, and V2 identified 914 835 (12.7%) as having technology codes compared with 805 585 (11.2%) by V3. The 2 systems were similar in accounting for the number of CCC body-system categories per patient and in explaining variation in hospital length of stay and in-hospital mortality. For both V2 and V3, 10.0% of the variance in hospital length of stay and 12.0% of the variance in in-hospital mortality was explained by the presence of a CCC. Similar patterns were observed when analyzing the 2 999 420 Medicaid Merative MarketScan Research Databases’ hospitalizations (52.3% of patients were male, the median age was 1 year [IQR, 0-12 years], and 62.0% were 0 to 4 years old), except that the percentages of identified CCCs were all lower: V2 identified 758 110 hospitalizations (25.3%) with any CCC compared with 718 100 (23.9%) identified by V3.

**Conclusions and Relevance:**

These results suggest that, moving forward, V3 should be used to identify CCCs, and ongoing, frequent updates to V3, using a transparent, structured process, will enable V3 to accurately reflect the evolving spectrum of clinical conditions represented in the *ICD-10-CM*.

## Key Points

**Question:**

How does the updated version of the pediatric complex chronic condition (CCC) system (version 3 [V3]) compare with version 2 (V2) in capturing the range of clinical conditions represented in the *International Statistical Classification of Diseases, Tenth Revision, Clinical Modification* (*ICD-10-CM*)?

**Findings:**

In this cross-sectional study of 7 186 019 hospitalizations within the Pediatric Health Information System from 2009 to 2019, V3 identified 38.3% of hospitalizations with a CCC compared with 40.1% identified by V2. The systems were similar in accounting for the number of CCC body-system categories per patient and in explaining variation in length of stay and mortality, and similar patterns were observed in the 2 999 420 hospitalizations from the Medicaid Merative MarketScan Research Databases.

**Meaning:**

The findings of this study suggest that the CCC V3 reflects the evolving *ICD-10-CM* system and should be used to identify CCCs.

## Teaser

This repeated cross-sectional study compares the updated version 3 pediatric complex chronic condition system with version 2 using hospitalization data from the Pediatric Health Information System and the Medicaid Merative MarketScan Research Databases from 2009 to 2019 for all patients aged 0 to 18 years.

## Introduction

In the US, the International Classification of Diseases (ICD) system with clinical modifications (ICD-CM) is used to code and classify diagnoses, symptoms, and procedures associated with health care utilization.^1,2^ The pediatric complex chronic condition (CCC) classification system uses ICD codes to determine the presence of 1 or more CCCs (10 body-system categories) and includes posttransplant and technology variables to signify additional complexity (Box).^3^ The open-source code CCC system is the most widely used classification system to study patients younger than 18 years with medical complexities. The CCC system has evolved alongside the ICD system and must account for updates in the underlying ICD codes: CCC version 1 (V1)^4^ corresponded to the *International Classification of Diseases, Ninth Revision, Clinical Modification* (*ICD-9-CM*) and version 2 (V2) to the transition to the *International Statistical Classification of Diseases, Tenth Revision, Clinical Modification* (*ICD-10-CM*).^3^ Since implementation of the *ICD-10-CM* in the US in 2015, however, thousands of new or related codes have been added to represent clinical conditions.^5^ The CCC system thus required a major update to capture the full spectrum of clinical conditions represented in the *ICD-10-CM*.

Box. **Complex Chronic Condition (CCC) System Version 3 Terminology and Definitions**

**General Terminology of the International Classification of Diseases–Clinical Modification (ICD-CM) System and Abbreviations**

**ICD**

System of disease classification maintained by the World Health Organization and updated approximately every 10 to 20 years. The current version is the *International Classification of Diseases, 11th Revision* (*ICD-11*).

**ICD-CM**

In the US, the Centers for Disease Control and Prevention’s National Center for Health Statistics, with authorization from the World Health Organization, issues an official modified version of the ICD system (ICD-CM). The current version is the *International Statistical Classification of Diseases, Tenth Revision, Clinical Modification* (*ICD-10-CM*) and is updated annually, chiefly by the addition of new codes.

**ICD Procedure Coding System (ICD-PCS)**

In the US, the Centers for Medicare & Medicaid Services since 2014 has provided codes for specific medical or surgical procedures; it is updated annually.

**Diagnosis (Dx)**

Codes in the ICD-CM system that specify disease conditions.

**Procedure (Pr)**

Codes in the ICD-PCS system that specify medical or surgical procedures.

**Technology (Tech)**

Codes in the ICD-CM or ICD-PCS system that specify use of a particular medical technology.

**General Terminology of the CCC System and Abbreviations**

**CCC**

Defined as “Any medical condition that can be reasonably expected to last at least 12 months (unless death intervenes) and to involve either several different organ systems or 1 organ system severely enough to require specialty pediatric care and probably some period of hospitalization in a tertiary care center.”^3,4^

**CCC Diagnostic (CCC-Dx) Codes**

ICD-CM Dx codes of conditions that conform to the CCC definition.

**CCC Procedure (CCC-Pr) Codes**

ICD-PCS Pr codes that imply that a patient has a condition that conforms to the CCC definition.

**CCC Technology (CCC-Tech) Codes**

ICD-CM Dx codes or ICD-PCS Pr codes indicating longer-term reliance on a specific implanted or invasive medical technology to maintain health.

**CCC Transplant (CCC-Txp) Codes**

ICD-CM Dx codes or ICD-PCS Pr codes indicating that the patient has undergone a solid organ or hematopoietic transplant that implies that a patient has a condition conforming to the CCC definition.

**CCC Categories**

Ten different categories of CCC conditions, conforming, for the most part, to the body-system organization of the ICD system but also conforming to some degree to medical subspecialities. The CCC categories are based on CCC-Dx, CCC-Pr, and CCC-Tech codes. The categories (listed in alphabetical order) are cardiovascular, congenital or genetic, gastrointestinal, hematologic or immunologic, malignancy, metabolic, neurologic and neuromuscular, premature and neonatal, renal and urologic, and respiratory.

**CCC-Tech Categories**

Seven different categories of technology, conforming, for the most part, to the body-system organization of the ICD system but also conforming to some degree to medical subspecialties. The categories (listed in alphabetical order) are cardiovascular tech, gastrointestinal tech, metabolic tech, miscellaneous tech, neuromuscular tech, renal tech, and respiratory tech.

**Patient-Specific Classification Variables of the CCC System and Abbreviations**

**CCC Status (CCC-Flag)**

For a given patient, the CCC status is positive if the patient has a CCC-Dx or -Pr code in the patient’s data record; if not, then the status is negative. Patients who are CCC status positive may have higher levels of chronic medical complexity than patients who have a negative status.

**CCC Category Status (CCC-Cat-Flag)**

For a given patient whose CCC status is positive, separate indicator variables for each of the 10 CCC categories indicate whether the patient has a CCC in that category. While the CCC-Flag is based only on CCC-Dx and -Pr codes, the CCC-Cat-Flag for each category is also based on CCC-Tech codes. This variable enables analyses of (or by) patients with different categories of CCCs.

**Number of CCC Categories (n-CCC)**

For a given patient whose CCC status is positive, this is the cumulative count of the CCC-Cat-Flags, which is to say the total number of CCC categories of conditions that the patient has, as indicated by the patient’s data record. Patients with higher values of the n-CCC count variable may have higher degrees of chronic medical complexity.

**CCC Technology Status (CCC-Tech-Flag)**

For a given patient whose CCC status is positive, this is an indicator variable regarding whether the patient’s data record includes a CCC-Tech code. This flag suggests that a patient has an additional degree of chronic medical complexity.

**CCC Technology Category Status (CCC-Tech-Cat-Flag)**

For a given patient whose CCC status is positive, these are separate indicator variables for each of the 7 CCC technology categories indicating whether the patient has technology in that category.

**CCC Transplant Status (CCC-Txp-Flag)**

For a given patient whose CCC status is positive, this is an indicator variable regarding whether the patient’s data record includes a CCC-Txp code. This flag suggests that a patient has an additional degree of chronic medical complexity.

For this update from CCC V2 to V3, we sought to address 4 additional important issues. First, we needed to update the CCC V3 system with new, missing, or retired codes to reflect the evolving *ICD-10-CM* system. A substantial amount of manual review was required to transition the CCC system from V1 to V2 because of the fundamentally different code formats and structures of the *ICD-9*, *ICD-9-CM*, *ICD-10*, and *ICD-10-CM*. More recently, the Centers for Medicare & Medicaid Services (CMS) began to regularly release digital *ICD-10-CM* update lists and comprehensive mapping files, which raised the possibility of using a semiautomated review process to identify new or missing codes.^5^ Second, because implanted medical technological support (hereinafter technology) is becoming more common and may not indicate a level of complexity consistent with the CCC operational definition, technology codes alone may not reliably identify CCC status (eg, the presence of a gastrostomy tube in a patient younger than 18 years with nutritional concerns but no additional medical complexity). We thus disaggregated CCC status from the presence of technology. A positive CCC status now depends on CCC diagnosis or procedure codes, while a separate technology variable indicates additional medical complexity. Third, we aimed to improve the transparency of decisions to include or exclude codes by creating open-source documentation of all CCC code determinations and changes. Finally, to address whether the change from V2 to V3 would alter the study results, we needed to compare the 2 versions.

In this study, we aimed to (1) describe the process used to update the CCC V3 system with new, missing, or retired codes (2) describe a new strategy of handling technology codes, and (3) compare the 2 versions using 2 inpatient pediatric administrative datasets.

## Methods

This cross-sectional study was deemed exempt from review by the Colorado Multiple Institutional Review Board because it did not constitute human participants research and used deidentified data; the requirement for informed consent was therefore waived. This study followed the Strengthening the Reporting of Observational Studies in Epidemiology (STROBE) reporting guideline.^6^

### Identification of New or Missing *ICD-10-CM* Codes

We used a comprehensive, supervised semiautomated strategy to identify new, missing, or retired *ICD-10-CM* codes. First, we remapped the previously published CCC V2 *ICD-9-CM* and *ICD-10-CM* codes using the 2018 General Equivalence Mappings electronic mapping tool from the CMS.^5^ All CCC V2 *ICD-9-CM* codes were forward remapped, and all CCC V2 *ICD-10* codes were remapped backward using the General Equivalence Mappings, which allowed for identification of new or missing codes. Second, we manually compared the remapped codes with annual update lists of new *ICD-10-CM* codes for 2019 to 2023 using the hierarchical *ICD-10* code structure to identify related codes that might represent a CCC.^5^ Third, 2 complex care pediatricians (J.A.F. and C.F.) independently reviewed all identified candidate codes for inclusion or exclusion in the CCC V3 system based on the CCC definition used to create the previous versions: “Any medical condition that can be reasonably expected to last at least 12 months (unless death intervenes) and to involve either several different organ systems or 1 organ system severely enough to require specialty pediatric care and probably some period of hospitalization in a tertiary care center.”^3,4^ Discrepancies between reviewers were resolved by joint review of the code or codes in question. Fourth, the final list of CCC V3 codes was created, reviewed for quality, and finalized for use. To enable transparency of code determinations, we created a list of all codes reviewed for inclusion or exclusion for CCC V3 and their determinations, including retired codes that were used in previous CCC versions (eTable 1 in Supplement 1, eTable 2 in Supplement 2, and eTable 3 in Supplement 3).

### Transplant and Technology Codes

In CCC V2, certain transplant codes and technology codes were used both to identify CCCs within the 10 CCC categories and to indicate additional medical complexity using transplant and technology variables. Whereas V3 implements CCC transplant codes identically to the V2 system, V3 implements CCC technology codes differently than the V2 system. This is because, unlike the transplant codes (which always signify end-organ failure corresponding to a CCC category), many new technology codes do not indicate body-system dysfunction and do not necessarily signify medical complexity. Accordingly, for V3, we separated CCC diagnosis and procedure codes from all CCC technology codes.

### Assignment of CCC Status and CCC Body-System Type

**Figure 1.**
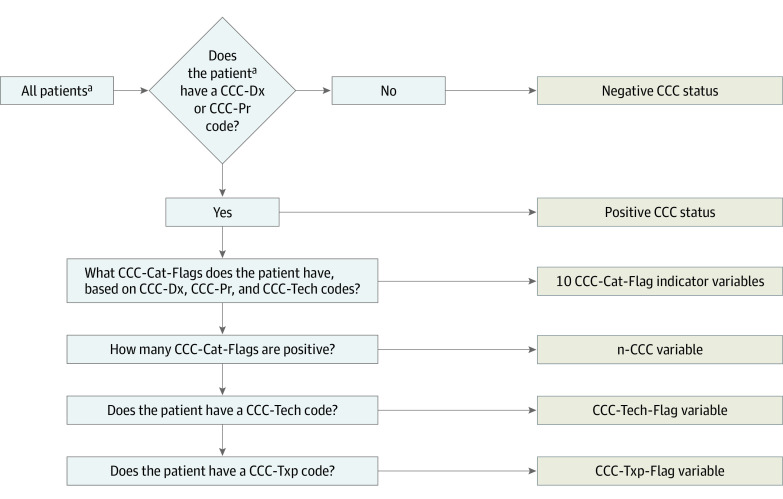
Process to Assign Complex Chronic Condition (CCC) Status and CCC Categories Using the CCC System Version 3 The CCC status (ie, yes or no) and CCC categories using CCC diagnosis, procedure, technology, and transplant codes. Cat indicates category; Dx, diagnosis; Flag, status; n-CCC, number of CCC categories; Pr, procedure; Tech, technology; Txp, transplant. ^a^Assigned at the patient level or encounter level (ie, a hospitalization).

Because of the changes to the handling of CCC technology codes, we reconceptualized how CCC status (ie, yes or no) and CCC category were assigned using a multistage process (Figure 1). The major difference between this CCC assignment strategy and prior versions is that in V3, to be classified as having a CCC, the patient (or encounter) must have a positive CCC status based only on the CCC diagnosis or procedure codes (and not based only on CCC technology codes). First, we assessed whether the patient had at least 1 CCC diagnosis or procedure code. If yes, the patient was classified as having a positive CCC status. Next, for patients with a positive CCC status, we assessed for the presence of technology using the technology codes. Finally, we identified the CCC categories that the patient had using CCC diagnostic, procedure, and technology codes.

### Comparison of the CCC V2 and V3 Classification Systems

We compared the CCC V2 and V3 systems using encounter-level data from the Pediatric Health Information System (PHIS) of the Children’s Hospital Association and the Medicaid Merative MarketScan Research Databases (hereinafter referred to as Medicaid) between January 1, 2009, to December 31, 2019. Both contain *ICD-9-CM* and *ICD-10-CM* diagnosis and procedure codes necessary for the analysis; PHIS includes up to 41 diagnosis and 41 procedure codes per encounter, and Medicaid includes up to 15 diagnosis and 15 procedure codes per encounter. All inpatient and observation encounters for patients 0 to 18 years old were included, except for “normal newborns” (MS-DRG 795) and patients who were pregnant (MS-MDC 14), according to the *ICD-10-CM* codes. Over the 11-year period, we compared (1) the percentage of patients identified with 1 or more body-system CCCs; (2) the percentage of patients by numbers of body-system CCCs per patient; and (3) the explanatory power of the CCC systems, as measured by the *R*^2^ value for length of inpatient stay and the pseudo-*R*^2^ value for in-hospital mortality. Patient race and ethnicity categories included Hispanic, non-Hispanic Black, non-Hispanic White, and other (including American Indian, Asian, Native Hawaiian, multiracial, other race, and missing). To enable comparisons between CCC V3 and previously published studies using the CCC system, race and ethnicity, as recorded in the PHIS and Medicaid databases, were reported.

### Statistical Analysis

All CCC lookup tables were created as Microsoft Excel comma-separated value spreadsheets to enable compatibility with an end user’s preferred database or statistical software. These lookup tables are open-source and available from the Children’s Hospital Association.^7^ Descriptive statistics and temporal graphs were used to compare the CCC V2 with the V3 systems. To assess relative changes for each CCC category from V2 to V3, percentage change was calculated using the following formula: 100 × (number of V3 CCC hospitalizations − number of V2 CCC hospitalizations)/number of V2 CCC hospitalizations. The McNemar test was used to compare differences in percentages between V2 and V3 by CCC category. Annual linear regressions for V2 and V3 were fit with the presence of a CCC as the only covariate and log-transformed length of stay (due to nonnormality) in days as the outcome. The *R*^2^ value and the Akaike information criterion (AIC) were used as the measures of fit for these models. Second, annual logistic regressions for V2 and V3 were fit with the presence of a CCC as the only covariate and mortality as the outcome. The McFadden pseudo-*R*^2^ and the AIC were used as the measures of fit. Data were analyzed from March 1, 2023, to April 1, 2024. All analyses were conducted using SAS, version 9.4 (SAS Institute Inc), and significance was set at a 2-tailed *P* < .05.

## Results

Overall, PHIS contained 7 186 019 hospitalizations (45.7% of patients were female, and 54.3% were male; the median age was 4 years [IQR, 1-11 years]), and Medicaid contained 2 999 420 hospitalizations (47.7% of patients were female, and 52.3% were male; the median age was 1 year [IQR, 0-12 years]). In PHIS, 51.2% of patients were between 0 and 4 years old and in Medicaid, 62.0% were included in this age group (the age range for both databases was 0 to 18 years). Of the total patients from the PHIS database, 19.4% were Hispanic, 19.0% were non-Hispanic Black, 49.0% were non-Hispanic White, and 12.5% were in the other race and ethnicity category. Among those patients from the Medicaid database, 6.1% were Hispanic, 28.5% were non-Hispanic Black, 43.3% were non-Hispanic White, and 22.1% were in the other race and ethnicity category. Additional demographics for both datasets are listed in eTable 4 in Supplement 1.

### Percentage of Patients Classified With CCCs, by CCC Status, CCC Category, and Technology Use

The percentages of hospitalizations with CCCs classified by V2 and V3 are listed in the Table. The CCC V2 identified 2 878 476 (40.1%) of the 7 186 019 encounters within PHIS as having any CCC compared with 2 753 412 (38.3%) identified by V3 (−4.3% change). Similarly, V2 identified 100 065 (1.4%) patients with transplant status compared with 146 683 (2.0%) by V3 (46.6% change). Finally, V2 identified 914 835 patients (12.7%) as having technology codes compared with 805 585 (11.2%) by V3 (−11.9% change). The Table contains the overall V2 results by CCC category and reports the V3 results based only on the presence of a CCC diagnosis or procedure code, excluding technology codes. The CCC categories (listed verbatim in alphabetical order) include any CCC, cardiovascular disease, congenital or genetic, gastrointestinal, hematologic or immunologic, malignancy, metabolic, neonatal, neuromuscular, renal, respiratory, tech dependence, and transplant. Comparing these data, several CCC categories remained stable (eg, malignancy: V2, 504 072 [7.0%] vs V3, 507 283 [7.1%]), while others demonstrated larger differences (eg, gastrointestinal: V2, 741 298 [10.3%] vs V3, 256 313 [3.6%]). The Table also reports V3 technology codes by CCC category among patients with a positive CCC status. For example, among any of the 7 186 019 total encounters, 422 072 (5.9%) had a positive CCC status and technology codes attributed to the gastrointestinal category. Finally, the Table reports the overall V3 results by CCC category for patients with a positive CCC status and technology codes present for that category. For example, 256 313 (3.6%) had either a gastrointestinal CCC diagnostic or procedure code, 422 072 (5.7%) had a gastrointestinal technology code, and a total of 678 385 (9.4%) were categorized by V3 as having a positive CCC status and gastrointestinal technology codes. Similar patterns were observed when analyzing Medicaid data (eTable 5 in Supplement 1), except that the percentages of identified CCCs were all lower in magnitude (V2 identified 758 113 [25.3%] hospitalizations with any CCC compared with 718 100 [23.9%] identified by V3).

**Table.** Patients With CCCs Classified by CCC V2 vs CCC V3 in the PHIS Database From 2009 to 2019^a^

CCC category	Patients with CCCs, No. (%) (N = 7 186 019)				Percentage Change^c^
	V2	V3^b^			
		Dx and Pr codes only	Tech codes only	Dx, Pr, and Tech codes	
Neuromuscular	698 224 (9.7)	711 683 (9.9)	12 625 (0.2)	724 308 (10.1)	3.7
Cardiovascular disease	617 572 (8.6)	599 123 (8.3)	29 389 (0.4)	628 512 (8.7)	1.8
Respiratory	323 756 (4.5)	277 532 (3.9)	52 517 (0.7)	330 049 (4.6)	1.9
Renal	314 510 (4.4)	258 457 (3.6)	47 333 (0.7)	305 790 (4.3)	−2.8
Gastrointestinal	74 1298 (10.3)	256 313 (3.6)	422 072 (5.9)	678 385 (9.4)	−8.5
Hematologic or immunologic	398 825 (5.6)	402 168 (5.6)	0	402 168 (5.6)	0.8
Metabolic	328 436 (4.6)	328 585 (4.6)	4906 (0.1)	333 491 (4.6)	1.5
Congenital or genetic	462 257 (6.4)	413 288 (5.8)	0	413 288 (5.8)	−10.6
Malignancy	504 072 (7.0)	507 283 (7.1)	0	507 283 (7.1)	0.6
Neonatal	197 444 (2.7)	181 040 (2.5)	0	181 040 (2.5)	−8.3
Transplant	100 065 (1.4)	145 864 (2.0)	819 (0)	146 683 (2.0)	46.6
Any CCC	2 878 476 (40.1)	2 753 412 (38.3)	NA	2 753 412 (38.3)	−4.3
Tech dependence	914 835 (12.7)	NA	805 585 (11.2)	NA	−11.9

Abbreviations: CCC, complex chronic condition; Dx, diagnosis; NA, not applicable; PHIS, Pediatric Health Information System Database; Pr, procedure; Tech, technology; V2, version 2; and V3, version 3.

^a^ Data include inpatient and observational hospitalizations.

^b^ Must have a positive CCC Flag (ie, status) variable.

^c^ Calculated by (V3 Dx, Pr, and Tech codes column – V2 column/V2 column) × 100. The McNemar test was used to compare differences in percentages between V2 and V3 by CCC category, which were all significant at *P* < .001.

### Numbers of CCCs per Patient Over Time

**Figure 2.**
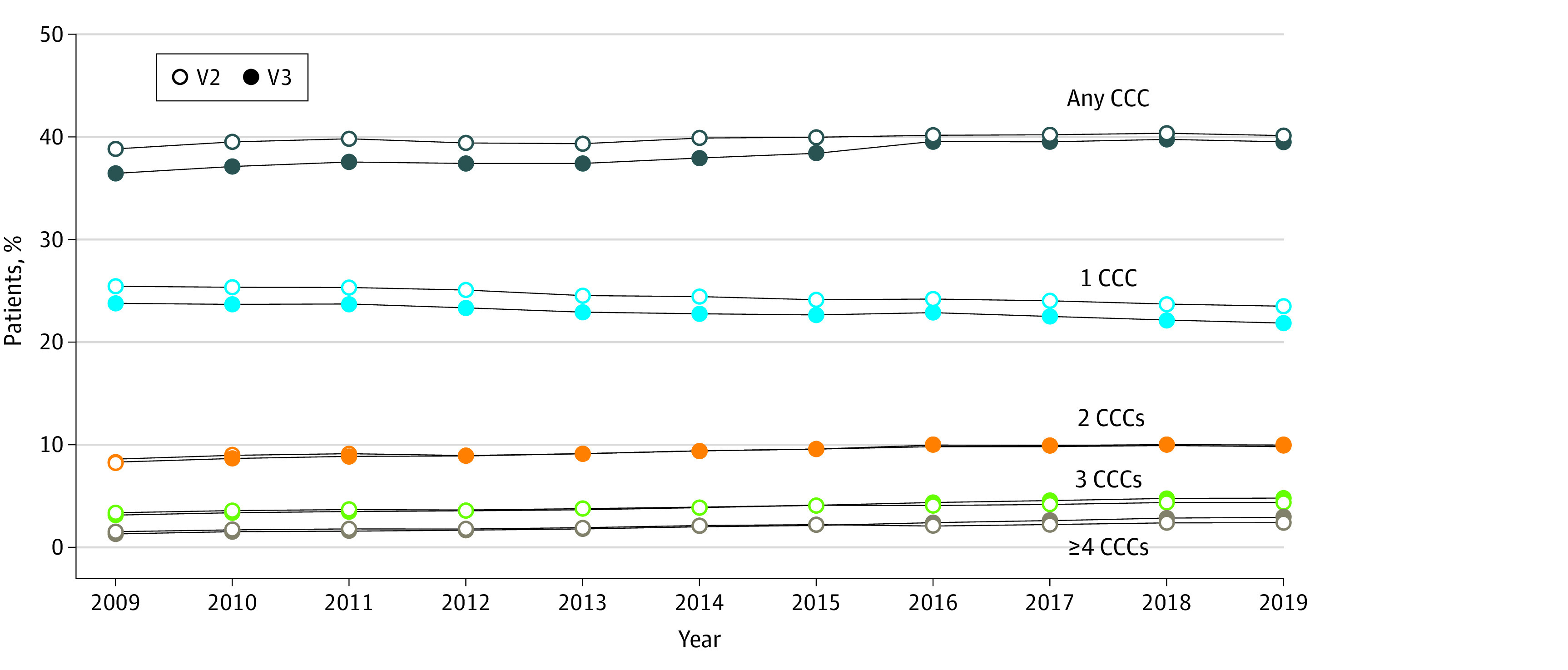
Percentage of Patients With Complex Chronic Conditions (CCCs) in the Pediatric Health Information System Database, by Year The McNemar test was used to compare differences in percentages between any CCC in version 2 (V2) and version 3 (V3) by year, which were all significant at *P* < .001.

Figure 2 displays the percentage of hospitalizations within the PHIS database with any CCC, 1 CCC, and multiple CCCs over time, as classified by either V2 or V3. The CCC V2 consistently identified a higher percentage of patients with a CCC compared with V3, although the difference was smaller after 2015. A subanalysis of the most recent year of PHIS data (eFigure 1 in Supplement 1) demonstrates how *ICD-10* code additions, deletions, and the reconceptualization of technology codes affected the observed net difference between V2 and V3. For both V2 and V3, the percentage of patients with 1 CCC decreased over time, although the percentages were consistently higher in V2 compared with V3. The latter was due to removal of nonspecific codes (eg, scoliosis, unspecified, other problems with newborn, and gastrostomy status) that were not alone indicative of a CCC. For example, removal of the diagnosis code Z931 (for gastrostomy status) in V3 resulted in 38 499 fewer CCC cases in a single year of data. Of those, 34 649 (90.0%) cases were still identified as having a CCC by a different V3 diagnosis code, resulting in 3850 cases no longer classified with a CCC. For both V2 and V3, the percentage of patients with multiple CCCs increased over time, and the percentage of hospitalizations with 3 CCCs and 4 or more CCCs was higher for V3 compared with V2 after 2015 (when the CMS stopped updating the *ICD-9-CM* and started releasing additional new *ICD-10-CM* codes that were only available for inclusion in V3 but not in V2). Similar patterns were observed when analyzing Medicaid data (eFigure 2 in Supplement 1).

### CCC-Associated Hospital Length of Stay and In-Hospital Mortality

**Figure 3.**
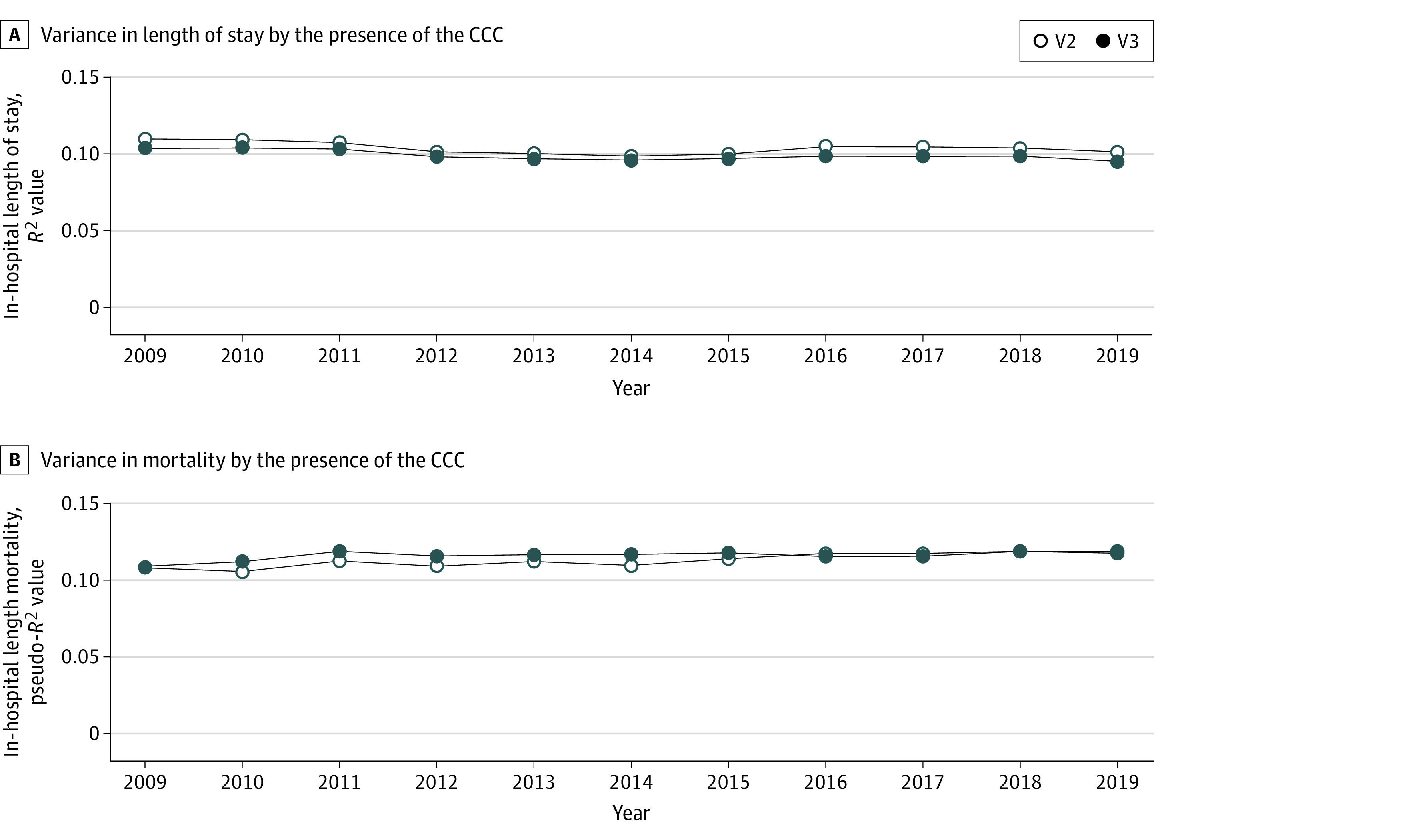
Comparison of the Complex Chronic Condition (CCC) Systems Version 2 (V2) and Version 3 (V3) in the Pediatric Health Information System Database by the Variance in Hospital Length of Stay and Mortality, by Year

For both V2 and V3, 10.0% of the variance in hospital length of stay within the PHIS database was explained by the presence of a CCC (Figure 3A). The *R*^2^ value for length of stay remained stable over time, although the values were consistently higher for V2 than V3. For both V2 and V3, 12.0% of the variance in in-hospital mortality was explained by the presence of a CCC (Figure 3B). The pseudo-*R*^2^ value for mortality increased slightly over time for both V2 and V3. The values were higher between 2009 and 2015 for V3 than for V2 and then converged after 2015. eTable 6 in Supplement 1 reports the AIC fit statistics for both models. Similar patterns were observed in Medicaid data (eFigure 3 in Supplement 1).

## Discussion

In this repeated cross-sectional study, CCC V2 identified 40.1% of the 7 186 019 patients within PHIS as having any CCC compared with 38.3% of the 7 186 019 patients identified by V3. Similarly, V2 identified 1.4% of patients with transplant status compared with 2.0% by V3, and V2 identified 12.7% of patients as having technology codes compared with 11.2% by V3. Over the 11-year study period, V2 consistently identified a higher percentage of patients with a CCC compared with V3, although the difference was smaller after full implementation of the *ICD-10-CM* in 2015. The 2 systems were comparable in accounting for the number of CCC body-system categories per patient and in explaining variation in inpatient length of stay and in-hospital mortality. Similar patterns were observed when analyzing Medicaid data, except that the percentages of identified CCCs were lower in magnitude (eg, 38.3% of patients in PHIS had a CCC using V3 vs 23.9% in Medicaid). Overall, CCC V3 and V2 were comparable across all outcome measures, and the updated CCC V3 system should be used to identify CCCs.

Minor differences between the 2 systems raise questions that deserve discussion. Why did V2 consistently identify a higher percentage of patients with CCCs than did V3? Why did this gap shrink after 2015? To address the first question, we must consider code deletions. In creating V3, we removed *ICD-10-CM* codes present in V2 that, when used alone, are not indicative of CCCs, such as scoliosis, unspecified (code M419), or other problems with newborn (code P84). The removal of these common nonspecific *ICD-10-CM* codes and corresponding pre-2015 *ICD-9* codes from V3 explains the sustained differences over time, including the pattern of V2 identifying a higher percentage of patients with 1 CCC than that of V3. For the second question, we must consider code additions. After 2015, the CMS stopped updating the *ICD-9-CM* and started releasing additional new *ICD-10-CM* codes that were available for inclusion in V3 (but not V2). For example, many new *ICD-10-CM* codes corresponding to stem cell transplants and organ transplant complications were added after 2015. However, many of these codes could not be reverse mapped to corresponding pre-2015 *ICD-9* codes, meaning that identification of patients with these CCC codes could only happen after 2015. These types of code additions likely explain the relative increase of patients with a CCC identified by V3 after 2015 by the increases in patients with multiple CCCs after 2015. The net result, therefore, of the transition from V2 to V3 is that slightly fewer patients younger than 18 years with only 1 CCC or only a technology-dependent CCC in V2 were classified as having a CCC in V3, whereas those with more than 1 CCC were present in nearly identical percentages.

These small differences notwithstanding, 2 aspects of the CCC V3 system warrant highlighting. First, end users must consider how to implement the CCC V3 system to meet their analytic needs in the context of the changes to V3. Some body-system categories may perform differently due to relocations of diagnoses within the CCC system. For example, the increase in neuromuscular CCCs was mostly attributable to the movement of cerebral infarction codes (433 and I63) from the cardiovascular to the neuromuscular CCC type. Similarly, the decrease in congenital or genetic CCCs was due to greater specificity of the included CCC codes and removal of nonspecific codes like constitutional short stature (E34.31). End users must also be aware that technology codes no longer are used to assign CCC status; instead, positive CCC status is based on the presence of CCC diagnosis or procedure codes only. Due to these changes in how technology codes are handled, some differences are immediately apparent, like the shift in the gastrointestinal CCC category by uncoupling CCC status from the presence of a gastrostomy tube.

Second, the CCC V3 system will require ongoing curation and maintenance, as ICD codes have been added, modified, or deleted from the overall *ICD-10-CM* system, and end users provided feedback after implementing CCC V3 for use in clinical and research settings. With this update, we moved from a CCC code identification process based entirely on manual identification and review to a transparent semiautomated identification and review process. With future updates, we anticipate moving toward a more automated process that leverages objective data-driven thresholds for identifying and including CCC codes. This process may be further enabled by the World Health Organization’s rollout of the *ICD-11*, which is a fully digital ICD system with linkages to medical terminology systems that are already highly integrated with clinical electronic health record systems.^2^

### Limitations

Our study must be considered in the context of 2 limitations. First, while we compared the systems using 2 common pediatric data sources (PHIS and Medicaid), we only analyzed inpatient hospitalization data. The CCC system could perform differently when applied to other data sources representing other settings of care (eg, Medicaid claims data for outpatient or emergency care settings) and depending on the number of diagnostic and procedure codes available per encounter. Second, if history is a guide, a limited number of specific codes will either have been included in or excluded from the CCC system by what will come to be viewed as errors in judgment. Moving toward a transparent system of informing users about code inclusion and exclusion decisions and incorporation of subsequent end user feedback will be extremely important to continued definitional calibration and improvement of the CCC system.

## Conclusions

In this cross-sectional study using 2 large databases of hospitalizations to compare CCC V2 with CCC V3, within PHIS, CCC V3 identified 38.3% as having any CCC vs with 40.1% by V2. The updated V3 system reflects the evolving *ICD-10-CM* system, and V3 should be used to identify CCCs. Ongoing frequent updates to V3 using a transparent structured process will enable V3 to accurately reflect the evolving spectrum of clinical conditions represented by the ICD system.

## Notes

Supplement 1. **eTable 1.** V3 CCC Categories and *ICD-9* and *ICD-10* Diagnosis and Procedure Codes **eTable 4.** Demographics of Patients With Hospitalizations in the Pediatric Health Information System and MarketScan Medicaid Databases (2009-2019) **eTable 5.** Percentage of Patients With CCCs Classified by CCC V2 vs CCC V3 in the Medicaid MarketScan Database (2009-2019) **eTable 6.** AIC Fit Statistics for Length of Stay and In-Hospital Mortality Regression Models by CCC V2 vs CCC V3 Using Pediatric Health Information System Data (2009-2019) **eFigure 1.** The Effects of CCC Version 3 *ICD-10* Code Additions, Deletions, and the Reconceptualization of Technology Codes on the Assignment of CCC Status for Hospitalizations in the Pediatric Health Information System (2019) **eFigure 2.** Percentage of Patients With CCCs in the MarketScan Medicaid Database (2009-2019) **eFigure 3.** Comparison of the CCC Systems V2 and V3 in the MarketScan Medicaid Database (2009-2019)

Supplement 2. **eTable 2.** CCC Version 3 Complete Code List

Supplement 3. **eTable 3.** CCC Version 3 Complete Code List With Deleted Items

Supplement 4.
Data Sharing Statement
