# Supplementary material for: Pediatric Complex Chronic Condition System Version 3
Source: JAMA Netw Open. 2024 Jul 15;7(7):e2420579. doi: 10.1001/jamanetworkopen.2024.20579 (PMC11250371; doi:10.1001/jamanetworkopen.2024.20579)
Supplement: Supplement 1. — eTable 1. V3 CCC Categories and ICD-9 and ICD-10 Diagnosis and Procedure Codes eTable 4. Demographics of Patients With Hospitalizations in the Pediatric Health Information System and MarketScan Medicaid Databases (2009-2019) eTable 5. Percentage of Patients With CCCs Classified by CCC V2 vs CCC V3 in the Medicaid MarketScan Database (2009-2019) eTable 6. AIC Fit Statistics for Length of Stay and In-Hospital Mortality Regression Models by CCC V2 vs CCC V3 Using Pediatric Health Information System Data (2009-2019) eFigure 1. The Effects of CCC Version 3 ICD-10 Code Additions, Deletions, and the Reconceptualization of Technology Codes on the Assignment of CCC Status for Hospitalizations in the Pediatric Health Information System (2019) eFigure 2. Percentage of Patients With CCCs in the MarketScan Medicaid Database (2009-2019) eFigure 3. Comparison of the CCC Systems V2 and V3 in the MarketScan Medicaid Database (2009-2019) [file jamanetwopen-e2420579-s001.pdf]

Supplemental Online Content

Feinstein JA, Hall M, Davidson A, Feudtner C. Pediatric complex chronic condition system version 3. *JAMA Netw Open*. 2024;7(7):e2420579.
doi:10.1001/jamanetworkopen.2024.20579

eTable 1. V3 CCC Categories and *ICD-9* and *ICD-10* Diagnosis and Procedure Codes
eTable 4. Demographics of Patients With Hospitalizations in the Pediatric Health Information System and MarketScan Medicaid Databases (2009-2019)
eTable 5. Percentage of Patients With CCCs Classified by CCC V2 vs CCC V3 in the Medicaid MarketScan Database (2009-2019)
eTable 6. AIC Fit Statistics for Length of Stay and In-Hospital Mortality Regression Models by CCC V2 vs CCC V3 Using Pediatric Health Information System Data (2009-2019)
eFigure 1. The Effects of CCC Version 3 *ICD-10* Code Additions, Deletions, and the Reconceptualization of Technology Codes on the Assignment of CCC Status for Hospitalizations in the Pediatric Health Information System (2019)
eFigure 2. Percentage of Patients With CCCs in the MarketScan Medicaid Database (2009-2019)
eFigure 3. Comparison of the CCC Systems V2 and V3 in the MarketScan Medicaid Database (2009-2019)

This supplemental material has been provided by the authors to give readers additional information about their work.

© 2024 Feinstein JA et al. *JAMA Network Open*.

eTable 1. V3 CCC Categories and *ICD-9* and *ICD-10* Diagnosis and Procedure Codes

	Mental retardation	318	F71, F72, F73
	CNS degeneration and diseases	330, 331.0, 331.1, 331.3, 331.4, 331.6, 331.7, 331.8, 331.9, 333.2, 334, 335, 336.1, 336.8, 337.9, 759.5	E75, F84.2, G11, G12, G31.0, G31.8, G31.9, G32.89, G90.9, G91.0, G91.1, G91.3, G91.4, G91.8, G91.9, G93.8, G93.9, G94, G95.19, G95.8, Q85.1
	Infantile cerebral palsy	343	G80
	Epilepsy	345.01, 345.11, 345.3, 345.41, 345.61, 345.71, 345.81, 345.91	G40.011, G40.019, G40.111, G40.119, G40.211, G40.219, G40.311, G40.319, G40.411, G40.419, G40.42, G40.803, G40.804, G40.813, G40.814, G40.823, G40.824, G40.833, G40.834. G40.911, G40.919, G40.A11, G40.A19, G40.B11, G40.B19
	Other neurologic disorders	341.8, 342, 344.00-344.09, 344.81, 344.9, 348.4, 780.03, **01.52, 01.53**	G36.1, G36.8, G36.9, G37, G81, G82.5, G83.5, G83.9, G93.1, G93.5, M41.4, M41.5, R40.3**, 0016070, 0016071, 0016072, 0016073, 0016074, 0016075, 0016076, 0016077, 0016078, 001607B, 0016370, 0016371, 0016372, 0016373, 0016374, 0016375, 0016376, 0016377, 0016378, 001637B, 001U074, 001U076, 001U077, 001U079, 001U374, 001U376, 001U377, 001U379, 00B70ZZ, 00B73ZZ, 00B74ZZ, 00T70ZZ, 00T73ZZ, 00T74ZZ**
	Occlusion of cerebral arteries	434.01, 434.11, 434.91	I63
	Muscular dystrophies and myopathies	359.0, 359.1, 359.21, 359.22, 359.23, 359.29, 359.3	G71, G72
	Movement diseases	332.0, 332.1, 333.0, 333.2, 333.4, 333.5, 333.7, 333.9	G10, G20, G21, G23, G24.02, G24.1, G24.2, G24.8, G25.5, G25.8, G25.9, G26, G80.3
**Cardiovascular**	Heart and great vessel malformations	745.0, 745.1, 745.2, 745.3, 745.6, 746, 747.1, 747.2, 747.3, 747.4, 747.81, 747.89, **35.8**	Q20, Q21.2, Q21.3, Q21.4, Q21.8, Q21.9, Q22, Q23, Q24, Q25.1, Q25.2, Q25.3, Q25.4, Q25.5, Q25.6, Q25.7, Q25.8, Q25.9, Q26, Q28.2, Q28.3, Q28.8, Q28.9 **021609Q, 02160JP, 02160JQ, 02160JR, 02160KP, 02160Z7, 02160ZP, 02163Z7, 021708S, 021708T, 021708U, 021709S, 021709T, 021709U, 02170AS, 02170AU, 02170JQ, 02170JS, 02170JU, 02170KS, 02170KU,02170ZP, 02170ZQ, 02170ZR,**

© 2024 Feinstein JA et al. *JAMA Network Open*.

			**02170ZS, 02170ZT, 02170ZU, 02173J6, 021K08P, 021K08Q, 021K08R, 021K09P, 021K0AP, 021K0AQ, 021K0JP, 021K0JQ, 021K0JR, 021K0KP, 021K0KQ, 021K0KR, 021K0ZP, 021K0ZQ, 021K0ZR, 021K0ZW, 021K4JP, 021K4KQ, 021L08R, 021L0KP, 021L0ZW, 024F07J, 024F08J, 024F0JJ, 024F0KJ, 024G072, 024G082, 024G0J2, 024G0K2, 024J072, 024J082, 024J0J2, 02BK0ZZ, 02LR0CT, 02LR0DT, 02LR0ZT, 02LS0ZZ, 02LT0ZZ, 02NH0ZZ, 02Q60ZZ, 02Q63ZZ, 02Q73ZZ, 02QA0ZZ, 02QA3ZZ, 02RK07Z, 02RK08Z, 02RK0JZ, 02RL0JZ, 02RM0JZ, 02RP0JZ, 02RQ07Z, 02RQ0JZ, 02RR07Z, 02RR0JZ, 02SP0ZZ, 02SW0ZZ, 02SX0ZZ, 02U607Z, 02U608Z, 02U60JZ, 02U707Z, 02U708Z, 02U70JZ, 02UA0JZ, 02UA3JZ, 02UA4JZ, 02UK07Z, 02UK08Z, 02UK0JZ, 02UK0KZ, 02UL07Z, 02UL0JZ, 02UL0KZ, 02UP07Z, 02UP08Z, 02UP0JZ, 02UP0KZ, 02UP38Z, 02UW07Z, 02UW08Z, 02UW0JZ, 02UW0KZ, 02UX07Z, 02UX08Z, 02UX0JZ, 02UX0KZ, 02UX3KZ, 02VR0ZT, 02W50JZ, 02WA0JZ, 02WM0JZ**
	Endocardium diseases	424.0, 424.2, 424.3	I34.0, I34.2, I34.8, I34.9, I36, I37
	Cardiomyopathies	425.0, 425.1, 425.2, 425.3, 425.4, 425.8, 429.1	I42, I43, I51.5
	Conduction disorder	426, 427.0, 427.1, 427.3, 427.4	I44, I45, I47, I48, I49.0
	Dysrhythmias	427.6, 427.8, 427.9	I49.1, I49.3, I49.4, I49.5, I49.8, I49.9, R00.1
	Other cardiovascular	416.1, 416.8, 416.9, 428.0, 428.1, 428.22, 428.23, 428.32, 428.33, 428.42, 428.43, 428.9, 429.3, 433.01, 433.11, 433.21, 433.31, 433.81, 433.91, 434.01, 434.11, 434.91, V45.81	I27, I50.20, I50.22, I50.23, I50.30, I50.32, I50.33, I50.40, I50.42, I50.43, I50.810, I50.812, I50.813, I50.814, I50.82, I50.83, I50.84, I50.89, I50.9, I51.7, I51.81, I63.0, I63.1, I63.2, Z95.1
	Transplant	996.83, V42.1, V42.2, V43.2, **37.51**	T86.2, T86.3, Z94.1, Z94.3 **02YA0Z0, 02YA0Z1, 02YA0Z2**
**Respiratory**	Respiratory malformations	748	Q30, Q31, Q32, Q33, Q34
	Chronic respiratory diseases	327.25, 416.0, 416.2, 516.30, 516.31, 516.37, 518.83, 518.84, 770.4, V45.76	G47.35, I27.0, I27.82, J84.1, J84.2, J84.8, J84.9, J96.1, J96.2, Z90.2
	Cystic fibrosis	277.0	E84
	Other respiratory	**30.3, 30.4, 32.4, 32.5**	**0B110Z4, 0B113Z4, 0B114Z4, 0BTC0ZZ, 0BTC4ZZ, 0BTD0ZZ, 0BTD4ZZ, 0BTF0ZZ, 0BTF4ZZ, 0BTG0ZZ, 0BTG4ZZ, 0BTJ0ZZ, 0BTJ4ZZ, 0BTK0ZZ, 0BTK4ZZ, 0BTL0ZZ, 0BTL4ZZ, 0BTM0ZZ, 0BTM4ZZ, 0CTS0ZZ, 0CTS4ZZ, 0CTS7ZZ, 0CTS8ZZ**
	Transplant	996.84, V42.6 **33.5, 33.6**	T86.3, T86.81, Z94.2, Z94.3 **0BYC0Z0, 0BYC0Z1, 0BYC0Z2, 0BYD0Z0, 0BYD0Z1, 0BYD0Z2, 0BYF0Z0, 0BYF0Z1, 0BYF0Z2, 0BYG0Z0, 0BYG0Z1, 0BYG0Z2,**

© 2024 Feinstein JA et al. *JAMA Network Open*.

			**0BYH0Z0, 0BYH0Z1, 0BYH0Z2, 0BYJ0Z0, 0BYJ0Z1, 0BYJ0Z2, 0BYK0Z0, 0BYK0Z1, 0BYK0Z2, 0BYL0Z0, 0BYL0Z1, 0BYL0Z2, 0BYM0Z0, 0BYM0Z1, 0BYM0Z2**
**Renal and Urologic**	Congenital anomalies	752.62, 753	Q60, Q61, Q62, Q63, Q64, Z87.732
	Chronic renal failure	585	N18
	Other renal/urologic	V45.73, V45.74, **55.5, 56.4, 56.71, 56.79, 57.7**	Z90.5, Z90.6, **0T160Z8, 0T160ZA, 0T164Z8, 0T164ZA, 0T170Z8, 0T170ZA, 0T174Z8, 0T174ZA, 0T180Z8, 0T180ZA, 0T184Z8, 0T184ZA, 0TB60ZZ, 0TB63ZZ, 0TB64ZZ, 0TB67ZZ, 0TB68ZZ, 0TB70ZZ, 0TB73ZZ, 0TB74ZZ, 0TB77ZZ, 0TB78ZZ, 0TT00ZZ, 0TT04ZZ, 0TT10ZZ, 0TT14ZZ, 0TT20ZZ, 0TT24ZZ, 0TT60ZZ, 0TT64ZZ, 0TT67ZZ, 0TT68ZZ, 0TT70ZZ, 0TT74ZZ, 0TT77ZZ, 0TT78ZZ, 0TTB0ZZ, 0TTB4ZZ, 0TTB7ZZ, 0TTB8ZZ, 0TTD0ZZ, 0TTD4ZZ, 0TTD7ZZ, 0TTD8ZZ**
	Chronic bladder diseases	344.61, 596.4, 596.53, 596.54	G83.4, N31
	Transplant	996.81, V42.0, **55.6**	T86.1, Z94.0 **0TY00Z0, 0TY00Z1, 0TY00Z2, 0TY10Z0, 0TY10Z1, 0TY10Z2**
**Gastrointestinal**	Congenital anomalies	750.3, 751.1, 751.2, 751.3, 751.4, 751.5, 751.6, 751.7. 751.8, 751.9	Q39.0, Q39.1, Q39.2, Q39.3, Q39.4, Q41, Q42, Q43.1, Q43.2, Q43.3, Q43.4, Q43.5, Q43.6, Q43.7, Q43.8, Q43.9, Q44, Q45, Z87.731
	Chronic liver disease and cirrhosis	571.4, 571.5, 571.6, 571.8, 571.9	K73, K74, K75.4, K76.0, K76.1, K76.2, K76.3, K76.5, K76.8
	Inflammatory bowel disease	555, 556	K50, K51
	Other gastrointestinal disease	453.0, 557.1, 560.2, 564.7, V45.3, V45.72, V45.75, **25.3, 25.4, 42.42, 43.9, 45.63, 45.8, 50.4, 52.6, 52.7, 54.71**	I82.0, K55.1, K56.2, K59.3, Z90.3, Z90.49, Z98.0 **0CT70ZZ, 0CT7XZZ, 0D13079, 0D1307A, 0D1307B, 0D1607A, 0D160ZA, 0DT50ZZ, 0DT54ZZ, 0DT57ZZ, 0DT58ZZ, 0DT60ZZ, 0DT64ZZ, 0DT67ZZ, 0DT68ZZ, 0DT80ZZ, 0DT84ZZ, 0DT87ZZ, 0DT88ZZ, 0DT90ZZ, 0DT94ZZ, 0DT97ZZ, 0DT98ZZ, 0DTE0ZZ, 0DTE4ZZ, 0DTE7ZZ, 0DTE8ZZ, 0FT00ZZ, 0FT04ZZ, 0FTG0ZZ, 0FTG4ZZ**
	Transplant	996.82, 996.86, 996.87, V42.7, V42.83, V42.84, **46.97, 50.5, 52.80, 52.82, 52.83, 52.84, 52.85, 52.86**	T86.4, T86.85, Z94.4, Z94.82, Z94.83 **0DY80Z0, 0DY80Z1, 0DY80Z2, 0DYE0Z0, 0DYE0Z1, 0DYE0Z2, 0FY00Z0, 0FY00Z1, 0FY00Z2, 0FYG0Z0, 0FYG0Z1, 0FYG0Z2, 3E030U0, 3E030U1, 3E033U0, 3E033U1, 3E0J3U0, 3E0J3U1, 3E0J7U0, 3E0J7U1, 3E0J8U0, 3E0J8U1**

© 2024 Feinstein JA et al. *JAMA Network Open*.

**Hematologic or immunologic**	Hereditary anemias	282.0, 282,1, 282.2, 282.3, 282.4, 282.5, 282.6	D55, D56, D57, D58
	Aplastic anemias	284	D60, D61, D71
	Hereditary immunodeficiency	279, 288.1, 288.2, 446.1	D72.0, D80, D81, D82, D83, D84, D89.40, D89.41, D89.42, D89.43, D89.49, D89.81, D89.82, D89.89, D89.9, M30.3, M35.9
	Coagulation/hemorrhagic	286.0, 286.3, 287.32, 287.33, 287.39	D66, D68.2, D69.4
	Leukopenia	288.01, 288.02	D70.0, D70.4
	Hemophagocytic syndromes	288.4	D76
	Sarcoidosis	135	D86
	Acquired immunodeficiency	042, V08	B20, Z21
	Polyarteritis nodosa and related conditions	446.0, 446.21, 446.4, 446.5, 446.6, 446.7	M30.0, M30.1, M30.2, M30.8, M31.0, M31.1, M31.3, M31.4, M31.5, M31.6, M31.7
	Diffuse diseases of connective tissue	710.0, 710.1, 710.3	M32.1, M32.8, M32.9, M33, M34
	Other hematologic/immunologic	**41.5**	**07TP0ZZ, 07TP4ZZ**
	Transplant	**41.0, 41.94**	**07YP0Z0, 07YP0Z1, 07YP0Z2, 30230AZ, 30230G0, 30230G1, 30230G2, 30230G3, 30230G4, 30230U2, 30230U3, 30230U4, 30230X0, 30230X1, 30230X2, 30230X3, 30230X4, 30230Y0, 30230Y1, 30230Y2, 30230Y3, 30230Y4, 30233AZ, 30233G0, 30233G1, 30233G2, 30233G3, 30233G4, 30233U2, 30233U3 30233U4, 30233X0, 30233X1, 30233X2, 30233X3, 30233X4, 30233Y0, 30233Y1, 30233Y2, 30233Y3, 30233Y4, 30240AZ, 30240G0, 30240G1, 30240G2, 30240G3, 30240G4, 30240U2, 30240U3, 30240U4, 30240X0, 30240X1, 30240X2, 30240X3, 30240X4, 30240Y0, 30240Y1, 30240Y2, 30240Y3, 30240Y4, 30243AZ, 30243G0, 30243G1, 30243G2, 30243G3, 30243G4, 30243U2, 30243U3, 30243U4, 30243X0, 30243X1, 30243X2, 30243X3, 30243X4, 30243Y0, 30243Y1, 30243Y2, 30243Y3, 30243Y4, 30250G0, 30250G1, 30250X0, 30250X1, 30250Y0, 30250Y1, 30253G0, 30253G1, 30253X0, 30253X1, 30253Y0, 30253Y1, 30260G0, 30260G1, 30260X0, 30260X1, 30260Y0, 30260Y1, 30263G0, 30263G1, 30263X0, 30263X1, 30263Y0, 30263Y1**
**Metabolic**	Amino acid metabolism	270	E70, E71, E72
	Carbohydrate metabolism	271	E74
	Lipid metabolism	272	E75, E77, E78, E88.1, E88.8
	Storage disorder	277.3, 277.5	E76, E85, M04.1
	Other metabolic disorders	275.0, 275.1, 275.2, 275.3, 277.2, 277.4, 277.6, 277.8, 277.9	D84.1, E79.1, E79.2, E79.8, E79.9, E80.3, E80.4, E80.5, E80.6, E80.7, E83.0, E83.1, E83.3, E83.4, E88.0, E88.2, E88.3, E88.4, E88.9, H49.81

© 2024 Feinstein JA et al. *JAMA Network Open*.

	Endocrine disorders	243, 253.2, 253.5, 253.6, 235.9, 255.0, 255.13, 255.2, **06.4, 06.52, 06.81, 07.3, 07.64, 07.65, 07.68, 07.69, 62.41, 62.42, 64.5, 65.5, 65.6, 68.4, 68.5, 68.6, 68.7**	E00, E03.0, E03.1, E22.2, E23.0, E23.2, E23.3, E23.7, E24.0, E24.2, E24.3, E24.8, E24.9, E25, E26.81, Z79.4, **0GT00ZZ, 0GT04ZZ, 0GT40ZZ, 0GT44ZZ, 0GTK0ZZ, 0GTK4ZZ, 0GTR0ZZ, 0GTR4ZZ, 0UT20ZZ, 0UT24ZZ, 0UT27ZZ, 0UT28ZZ, 0UT2FZZ, 0UT40ZZ, 0UT44ZZ, 0UT47ZZ, 0UT48ZZ, 0UT70ZZ, 0UT74ZZ, 0UT90ZZ, 0UT94ZZ, 0UT97ZZ, 0UT98ZZ, 0UT9FZZ, 0UTC0ZZ, 0UTC7ZZ, 0UTC8ZZ, 0VTC0ZZ, 0VTC4ZZ, 0W4M070, 0W4M0J0, 0W4M0K0, 0W4M0Z0, 0W4N071, 0W4N0J1, 0W4N0K1, 0W4N0Z1**
**Other Congenital or Genetic Defect**	Chromosomal anomalies	758	Q90, Q91, Q92, Q93, Q95.0, Q95.2, Q95.3, Q95.5, Q95.8, Q95.9, Q96, Q97, Q98, Q99.0, Q99.1, Q99.8, Q99.9
	Bone and join anomalies	259.4, 737.31, 737.32, 737.33, 756.0, 756.1, 756.2, 756.3, 756.4, 756.5	E34.3 (do not include E34.31), E34.30, E34.32, E34.9, M41.0, M43.3, M96.5, Q72.0, Q72.1, Q72.2, Q75, Q76.0, Q76.1, Q76.2, Q76.4, Q76.5. Q76.6, Q76.7, Q77, Q78.0, Q78.1, Q78.2, Q78.3, Q78.4, Q78.6, Q78.8, Q78.9
	Diaphragm and abdominal wall	553.3, 756.6, 756.7	K44.9, Q79.0, Q79.1, Q79.2, Q79.3, Q79.4, Q79.5, Q79.9, Z87.760, Z87.761, Z87.762
	Other congenital anomalies	759.7, 759.8, 759.9	Q81, Q87.0, Q87.11, Q87.2, Q87.3, Q87.4, Q87.8, Q89.7, Q89.8, Q89.9, Q99.2
**Malignancy**	Neoplasms	140-209, 230-239, **00.10, 99.25**	C00-C43, C4A, C44-C75, C7A, C7B, C76-C96, D00-D09, D3A.0, D37-D44, D45-D49, Q85.0, Q85.8 **3E00X05, 3E01305, 3E02305, 3E03005, 3E03305, 3E04005, 3E04305, 3E05005, 3E05305, 3E06005, 3E06305, 3E0A305, 3E0F305, 3E0F705, 3E0F805, 3E0G305, 3E0G705, 3E0G805, 3E0H305, 3E0H705, 3E0H805, 3E0J305, 3E0J705, 3E0J805, 3E0K305, 3E0K705, 3E0K805, 3E0L305, 3E0L705, 3E0M305, 3E0M705, 3E0N305, 3E0N705, 3E0N805, 3E0P305, 3E0P705, 3E0P805, 3E0Q305, 3E0Q705, 3E0R305, 3E0S305, 3E0V305, 3E0W305, 3E0Y305, 3E0Y705**
	Transplantation	996.85, V42.81, V42.82	T86.0, T86.5, Z94.81, Z94.84
**Premature and Neonatal**	Fetal malnutrition	764.01, 764.02, 764.11, 764.12, 764.21, 764.22, 764.91, 764.92	P05.01, P05.02, P05.11, P05.12, P05.2, P05.9
	Extreme immaturity	765.01, 765.02, 765.11, 765.12, 765.21, 765.22, 765.23	P07.01, P07.02, P07.21, P07.22, P07.23, P07.24, P07.25
	Cerebral hemorrhage at birth	767.0	P10.0, P10.1, P10.4, P10.8, P10.9, P52.21, P52.22, P52.4, P25.5, P25.6, P52.8, P25.9
	Spinal cord injury at birth	767.4	P11.5
	Birth asphyxia	768.5, 768.9	no ICD10 code
	Respiratory diseases	770.2, 770.7	P25, P27
	Hypoxic-ischemic encephalopathy	768.7	P91.6

© 2024 Feinstein JA et al. *JAMA Network Open*.

	Other premature or neonatal	771.0, 771.1, 772.13, 772.14, 773.3, 773.4, 774.7, 776.5, 777.53, 778.0, 779.7	P11.1, P11.2, P11.9, P35.0, P35.1, P35.4, P56.0, P56.9, P57, P61.3, P61.4, P77.3, P83.2, P91.2, Z87.61
**Device & Technology Usage**	Neurologic/Neuromuscular	349.1, 996.2, 996.63, V45.2, V53.01, V53.02 **02.2, 02.3, 02.4, 02.93, 03.7, 03.93, 03.97, 04.92**	T85.01XA, T85.02XA, T85.03XA, T85.09XA, T85.110A, T85.111A, T85.112A, T85.113A, T85.118A, T85.120A, T85.121A, T85.122A, T85.123A, T85.128A, T85.190A, T85.191A, T85.192A, T85.193A, T85.199A, T85.730A, T85.731A, T85.732A, T85.733A, T85.734A, T85.735A, T85.738A, T85.810A, T85.820A, T85.830A, T85.840A, T85.850A, T85.860A, T85.890A, Z45.41, Z45.42, Z45.49, Z96.82, Z98.2 **00160J0, 00160J1, 00160J2, 00160J3, 00160J4, 00160J5, 00160J6, 00160J7, 00160J8, 00160JB, 00160K0, 00160K1, 00160K2, 00160K3, 00160K4, 00160K5, 00160K6, 00160K7, 00160K8, 00160KB, 00163J0, 00163J1, 00163J2, 00163J3, 00163J4, 00163J5, 00163J6, 00163J7, 00163J8, 00163JB, 00163K0, 00163K1, 00163K2, 00163K3, 00163K4, 00163K5, 00163K6, 00163K7, 00163K8, 00163KB, 001U0J4, 001U0J6, 001U0J7, 001U0J9, 001U0K4, 001U0K6, 001U0K7, 001U0K9, 001U3J4, 001U3J6, 001U3J7, 001U3J9, 001U3K4, 001U3K6, 001U3K7, 001U3K9, 009600Z, 009630Z, 009640Z, 00H00MZ, 00H03MZ, 00H04MZ, 00H60MZ, 00H63MZ, 00H64MZ, 00HE0MZ, 00HE3MZ, 00HE4MZ, 00HU0MZ, 00HU3MZ, 00HU4MZ, 00HV0MZ, 00HV3MZ, 00HV4MZ, 00W60JZ, 00W63JZ, 00W64JZ, 00WU0JZ, 00WU3JZ, 00WU4JZ, 01HY0MZ, 01HY3MZ, 01HY4MZ, 0DH60MZ, 0DH63MZ, 0DH64MZ, 0W110J9, 0W110JB, 0W110JG, 0W110JJ, 3E1Q38X, 3E1Q38Z**
	Cardiovascular	996.0, 996.1, 996.61, 996.62, V43.3, V45.0, V53.3 **00.50, 00.51, 00.53, 00.54, 00.55, 00.57, 17.51, 17.52, 37.41, 37.52, 37.53, 37.54, 37.55, 37.60, 37.61, 37.63, 37.65, 37.66, 37.67, 37.71, 37.72, 37.74, 37.76, 37.79, 37.80, 37.81, 37.82, 37.83, 37.85, 37.86, 37.87, 37.89, 37.94, 37.95, 37.96, 37.97, 37.98, 39.81, 39.82, 39.83, 39.84, 39.85, 89.45, 89.46, 89.47, 89.48, 89.49**	T82.01XA, T82.02XA, T82.03XA, T82.09XA, T82.110A, T82.111A, T82.118A, T82.119A, T82.120A, T82.121A, T82.128A, T82.129A, T82.190A, T82.191A, T82.198A, T82.199A, T82.211A, T82.212A, T82.213A, T82.218A, T82.221A, T82.222A, T82.223A, T82.228A, T82.310A, T82.311A, T82.312A, T82.318A, T82.319A, T82.320A, T82.321A, T82.322A, T82.328A, T82.329A, T82.330A, T82.331A, T82.332A, T82.338A, T82.339A, T82.390A, T82.391A, T82.392A, T82.398A, T82.399A, T82.41XA, T82.42XA, T82.43XA, T82.49XA, T82.510A, T82.511A, T82.512A, T82.513A, T82.515A, T82.518A, T82.519A, T82.520A, T82.521A, T82.522A, T82.523A, T82.525A, T82.528A, T82.529A, T82.530A, T82.531A, T82.532A, T82.533A, T82.535A, T82.538A, T82.539A, T82.590A, T82.591A, T82.592A, T82.593A, T82.595A, T82.598A, T82.599A, T82.6XXA, T82.7XXA, T82.817A, T82.818A, T82.827A, T82.828A, T82.837A, T82.838A, T82.847A, T82.848A, T82.855A, T82.856A, T82.857A, T82.858A, T82.867A, T82.868A, T82.897A, T82.898A, T82.9XXA, Z45.10, Z45.018, Z45.02, Z45.09, Z95.0, Z95.2, Z95.3, Z95.4, Z95.8

© 2024 Feinstein JA et al. *JAMA Network Open*.

			**02H40JZ, 02H40KZ, 02H43JZ, 02H44JZ, 02H44KZ, 02H60JZ, 02H60KZ, 02H63JZ, 02H63KZ, 02H63MZ, 02H64JZ, 02H64KZ, 02H70KZ, 02H73JZ, 02H73KZ, 02H73MZ, 02H74KZ, 02HA0QZ, 02HA0RS, 02HA0RZ, 02HA3QZ, 02HA3RS, 02HA4QZ, 02HA4RS, 02HK0JZ, 02HK0KZ, 02HK3JZ, 02HK3KZ, 02HK3MZ, 02HK4JZ, 02HK4KZ, 02HL0JZ, 02HL0KZ, 02HL0MZ, 02HL3JZ, 02HL3KZ, 02HL3MZ, 02HL4JZ, 02HL4KZ, 02HL4MZ, 02HN0JZ, 02HN0KZ, 02HN0MZ, 02HN3JZ, 02HN3KZ, 02HN3MZ, 02HN4JZ, 02HN4KZ, 02HN4MZ, 02WA0QZ, 02WA0RZ, 02WA3QZ, 02WA3RZ, 02WA4QZ, 02WA4RZ, 03HK0MZ, 03HK3MZ, 03HK4MZ, 03HL0MZ, 03HL3MZ, 03HL4MZ, 03WY0MZ, 03WY3MZ, 03WY4MZ, 0JH600Z, 0JH605Z, 0JH606Z, 0JH607Z, 0JH608Z, 0JH609Z, 0JH60AZ, 0JH60MZ, 0JH60PZ, 0JH630Z, 0JH635Z, 0JH636Z, 0JH637Z, 0JH638Z, 0JH639Z, 0JH63AZ, 0JH63MZ, 0JH63PZ, 0JH70MZ, 0JH73MZ, 0JH800Z, 0JH805Z, 0JH806Z, 0JH807Z, 0JH808Z, 0JH809Z, 0JH80AZ, 0JH80MZ, 0JH80PZ, 0JH830Z, 0JH835Z, 0JH836Z, 0JH837Z, 0JH838Z, 0JH839Z, 0JH83AZ, 0JH83MZ, 0JH83PZ, 0JWT0MZ, 0JWT0PZ, 0JWT3MZ, 0JWT3PZ, 0JWTXMZ, 4B02XSZ, 4B02XTZ, 5A02110, 5A02116, 5A0211D, 5A02210, 5A02216, 5A0221D**
	Respiratory	519.0, V44.0, V46.0, V46.1, V55.0<br>**31.2, 31.41, 31.74, 33.21, 34.85, 96.55, 97.23**	J95.0, J95.850, Z43.0, Z93.0, Z99.0, Z99.11, Z99.12<br>**0B110F4, 0B113F4, 0B114F4, 0B21XFZ, 0BHR0MZ, 0BHR3MZ, 0BHR4MZ, 0BHS0MZ, 0BHS3MZ, 0BHS4MZ, 0BHT0MZ, 0BHT3MZ, 0BHT4MZ, 0BP1XFZ, 0BW10FZ, 0BW13FZ, 0BW14FZ, 0JH604Z, 0JH634Z, 0JH804Z, 0JH834Z, 0WQ6XZ2, 3E1F78Z**
	Renal/Urologic	996.68, V44.5, V44.6, V45.1, V53.6, V55.5, V55.6, V56<br>**38.95, 39.27, 39.42, 39.93, 39.94, 39.95, 54.98, 55.02, 55.03, 55.04, 55.12, 55.93, 55.94, 55.97, 56.5, 56.6, 56.72, 56.73, 56.74, 56.75, 57.2, 59.93, 59.94, 86.07, 96.45, 96.46, 96.47**	T85.71XA, Z43.5, Z43.6, Z49, Z91.15, Z93.5, Z93.6, Z99.2<br>**031209D, 031209F, 03120AD, 03120AF, 03120JD, 03120JF, 03120KD, 03120KF, 03120ZD, 03120ZF, 031309D, 031309F, 03130AD, 03130AF, 03130JD, 03130JF, 03130KD, 03130KF, 03130ZD, 03130ZF, 031409D, 031409F, 03140AD, 03140AF, 03140JD, 03140JF, 03140KD, 03140KF, 03140ZD, 03140ZF, 031509D, 031509F, 03150AD, 03150AF, 03150JD, 03150JF, 03150KD, 03150KF, 03150ZD, 03150ZF, 031609D, 031609F, 03160AD, 03160AF, 03160JD, 03160JF, 03160KD, 03160KF, 03160ZD, 03160ZF, 031709D, 031709F, 03170AD, 03170AF, 03170JD, 03170JF, 03170KD, 03170KF, 03170ZD, 03170ZF, 031809D, 031809F, 03180AD, 03180AF, 03180JD, 03180JF, 03180KD, 03180KF, 03180ZD, 03180ZF, 031909F, 03190AF, 03190JF, 03190KF, 03190ZF, 031A09F, 031A0AF, 031A0JF, 031A0KF, 031A0ZF, 031B09F, 031B0AF, 031B0JF, 031B0KF, 031B0ZF, 031C09F, 031C0AF, 031C0JF, 031C0KF, 031C0ZF, 03WY0JZ, 03WY3JZ, 03WY4JZ, 03WYXJZ, 05HY33Z, 06HY33Z, 0JH60WZ, 0JH60XZ, 0JH63WZ, 0JH63XZ, 0JH80WZ, 0JH80XZ,**

© 2024 Feinstein JA et al. *JAMA Network Open*.

			**0JH83WZ, 0JH83XZ, 0JHD0WZ, 0JHD0XZ, 0JHD3WZ, 0JHD3XZ, 0JHF0WZ, 0JHF0XZ, 0JHF3WZ, 0JHF3XZ, 0JHL0WZ, 0JHL0XZ, 0JHL3WZ, 0JHL3XZ, 0JHM0WZ, 0JHM0XZ, 0JHM3WZ, 0JHM3XZ, 0T130ZB, 0T134ZB, 0T140ZB, 0T144ZB, 0T16079, 0T1607C, 0T1607D, 0T160J9, 0T160JC, 0T160JD, 0T160K9, 0T160KC, 0T160KD, 0T160Z9, 0T160ZC, 0T160ZD, 0T163JD, 0T16479, 0T1647C, 0T1647D, 0T164J9, 0T164JC, 0T164JD, 0T164K9, 0T164KC, 0T164KD, 0T164Z9, 0T164ZC, 0T164ZD, 0T17079, 0T1707C, 0T1707D, 0T170J9, 0T170JC, 0T170JD, 0T170K9, 0T170KC, 0T170KD, 0T170Z9, 0T170ZC, 0T170ZD, 0T173JD, 0T17479, 0T1747C, 0T1747D, 0T174J9, 0T174JC, 0T174JD, 0T174K9, 0T174KC, 0T174KD, 0T174Z9, 0T174ZC, 0T174ZD, 0T18079, 0T1807C, 0T1807D, 0T180J9, 0T180JC, 0T180JD, 0T180K9, 0T180KC, 0T180KD, 0T180Z9, 0T180ZC, 0T180ZD, 0T183JD, 0T18479, 0T1847C, 0T1847D, 0T184J9, 0T184JC, 0T184JD, 0T184K9, 0T184KC, 0T184KD, 0T184Z9, 0T184ZC, 0T184ZD, 0T1B079, 0T1B07C, 0T1B07D, 0T1B0J9, 0T1B0JC, 0T1B0JD, 0T1B0K9, 0T1B0KC, 0T1B0KD, 0T1B0Z9, 0T1B0ZC, 0T1B0ZD, 0T1B479, 0T1B47C, 0T1B47D, 0T1B4J9, 0T1B4JC, 0T1B4JD, 0T1B4K9, 0T1B4KC, 0T1B4KD, 0T1B4Z9, 0T1B4ZC, 0T1B4ZD, 0T25X0Z, 0T29X0Z, 0T29XYZ, 0T2BX0Z, 0T9000Z, 0T9030Z, 0T9040Z, 0T9070Z, 0T9080Z, 0T9100Z, 0T9130Z, 0T9140Z, 0T9170Z, 0T9180Z, 0T9370Z, 0T9380Z, 0T9470Z, 0T9480Z, 0TQ67ZZ, 0TQ77ZZ, 3E1K38Z, 3E1M39Z, 5A1D60Z, 5A1D70Z, 5A1D80Z, 5A1D90Z**
	Gastrointestinal	536.4, V44.1, V44.2, V44.3, V44.4, V53.5, V55.1, V55.2, V55.3, V55.4<br>**42.10, 42.11, 42.81, 43.1, 44.12, 44.32, 44.38, 44.39, 46.13, 46.22, 46.23, 46.32, 46.40, 46.41, 46.43, 96.24, 96.36, 97.02**	K94.2, Z43.1, Z43.2, Z43.3, Z43.4, Z46.5, Z93.1, Z93.2, Z93.3, Z93.4<br>**0D11074, 0D110J4, 0D110K4, 0D110Z4, 0D113J4, 0D11474, 0D114J4, 0D114K4, 0D114Z4, 0D15074, 0D150J4, 0D150K4, 0D150Z4, 0D153J4, 0D15474, 0D154J4, 0D154K4, 0D154Z4, 0D16074, 0D160J4, 0D160J9, 0D160JA, 0D160K4, 0D160K9, 0D160KA, 0D160Z4, 0D163J4, 0D16474, 0D164J4, 0D164J9, 0D164JA, 0D164K4, 0D164K9, 0D164KA, 0D164Z4, 0D16874, 0D168J4, 0D168J9, 0D168JA, 0D168K4, 0D168K9, 0D168KA, 0D168Z4, 0D1B0Z4, 0D1B4Z4, 0D1B8Z4, 0D1H0Z4, 0D1H4Z4, 0D1H8Z4, 0D1K0Z4, 0D1K4Z4, 0D1K8Z4, 0D1L0Z4, 0D1L4Z4, 0D1L8Z4, 0D1N0Z4, 0D1N4Z4, 0D1N8Z4, 0D20X0Z, 0D20XUZ, 0D20XYZ, 0D787ZZ, 0D7E7ZZ, 0DBB7ZZ, 0DH50DZ, 0DH50UZ, 0DH53DZ, 0DH53UZ, 0DH54DZ, 0DH54UZ, 0DH57DZ, 0DH57UZ, 0DH58DZ, 0DH58UZ, 0DH63UZ, 0DH64UZ, 0DHA3UZ, 0DHA4UZ, 0DHA8UZ, 0DN87ZZ, 0DNE7ZZ, 0DW04UZ, 0DW08UZ, 0WQFXZ2, 3E1G78Z, 3E1H78Z**
	Metabolic	V45.85, V53.91, **86.06**	Z46.81, Z96.41

© 2024 Feinstein JA et al. *JAMA Network Open*.

			**0JH60VZ, 0JH63VZ, 0JH70VZ, 0JH73VZ, 0JH80VZ, 0JH83VZ, 0JHD0VZ, 0JHD3VZ, 0JHF0VZ, 0JHF3VZ, 0JHG0VZ, 0JHG3VZ, 0JHH0VZ, 0JHH3VZ, 0JHL0VZ, 0JHL3VZ, 0JHM0VZ, 0JHM3VZ, 0JHN0VZ, 0JHN3VZ, 0JHP0VZ, 0JHP3VZ, 0JHT0VZ, 0JHT3VZ**
	Miscellaneous	996.4, 996.66, 996.67, 996.9, V46.2, **81.0, 81.3, 84.51**	T87.0X9, T87.1X9, T87.2, Y83.1, Y83.3, Z99.81<br>**0RG0070, 0RG0071, 0RG007J, 0RG00A0, 0RG00A1, 0RG00AJ, 0RG00J0, 0RG00J1, 0RG00JJ, 0RG00K0, 0RG00K1, 0RG00KJ, 0RG00Z0, 0RG00Z1, 0RG00ZJ, 0RG0370, 0RG0371, 0RG037J, 0RG03A0, 0RG03A1, 0RG03AJ, 0RG03J0, 0RG03J1, 0RG03JJ, 0RG03K0, 0RG03K1, 0RG03KJ, 0RG03Z0, 0RG03Z1, 0RG03ZJ, 0RG0470, 0RG0471, 0RG047J, 0RG04A0, 0RG04A1, 0RG04AJ, 0RG04J0, 0RG04J1, 0RG04JJ, 0RG04K0, 0RG04K1, 0RG04KJ, 0RG04Z0, 0RG04Z1, 0RG04ZJ, 0RG1070, 0RG1071, 0RG107J, 0RG10A0, 0RG10A1, 0RG10AJ, 0RG10J0, 0RG10J1, 0RG10JJ, 0RG10K0, 0RG10K1, 0RG10KJ, 0RG10Z0, 0RG10Z1, 0RG10ZJ, 0RG1370, 0RG1371, 0RG137J, 0RG13A0, 0RG13A1, 0RG13AJ, 0RG13J0, 0RG13J1, 0RG13JJ, 0RG13K0, 0RG13K1, 0RG13KJ, 0RG13Z0, 0RG13Z1, 0RG13ZJ, 0RG1470, 0RG1471, 0RG147J, 0RG14A0, 0RG14A1, 0RG14AJ, 0RG14J0, 0RG14J1, 0RG14JJ, 0RG14K0, 0RG14K1, 0RG14KJ, 0RG14Z0, 0RG14Z1, 0RG14ZJ, 0RG2070, 0RG2071, 0RG207J, 0RG20A0, 0RG20A1, 0RG20AJ, 0RG20J0, 0RG20J1, 0RG20JJ, 0RG20K0, 0RG20K1, 0RG20KJ, 0RG20Z0, 0RG20Z1, 0RG20ZJ, 0RG2370, 0RG2371, 0RG237J, 0RG23A0, 0RG23A1, 0RG23AJ, 0RG23J0, 0RG23J1, 0RG23JJ, 0RG23K0, 0RG23K1, 0RG23KJ, 0RG23Z0, 0RG23Z1, 0RG23ZJ, 0RG2470, 0RG2471, 0RG247J, 0RG24A0, 0RG24A1, 0RG24AJ, 0RG24J0, 0RG24J1, 0RG24JJ, 0RG24K0, 0RG24K1, 0RG24KJ, 0RG24Z0, 0RG24Z1, 0RG24ZJ, 0RG4070, 0RG4071, 0RG407J, 0RG40A0, 0RG40A1, 0RG40AJ, 0RG40J0, 0RG40J1, 0RG40JJ, 0RG40K0, 0RG40K1, 0RG40KJ, 0RG40Z0, 0RG40Z1, 0RG40ZJ, 0RG4370, 0RG4371, 0RG437J, 0RG43A0, 0RG43A1, 0RG43AJ, 0RG43J0, 0RG43J1, 0RG43JJ, 0RG43K0,, 0RG43K1, 0RG43KJ, 0RG43Z0, 0RG43Z1, 0RG43ZJ, 0RG4470, 0RG4471, 0RG447J, 0RG44A0, 0RG44A1, 0RG44AJ, 0RG44J0, 0RG44J1, 0RG44JJ, 0RG44K0, 0RG44K1, 0RG44KJ, 0RG44Z0, 0RG44Z1, 0RG44ZJ, 0RG6070, 0RG6071, 0RG607J, 0RG60A0, 0RG60A1, 0RG60AJ, 0RG60J0, 0RG60J1, 0RG60JJ, 0RG60K0, 0RG60K1, 0RG60KJ, 0RG60Z0, 0RG60Z1, 0RG60ZJ, 0RG6370, 0RG6371, 0RG637J, 0RG63A0, 0RG63A1, 0RG63AJ, 0RG63J0, 0RG63J1, 0RG63JJ, 0RG63K0, 0RG63K1, 0RG63KJ, 0RG63Z0, 0RG63Z1, 0RG63ZJ, 0RG6470, 0RG6471, 0RG647J, 0RG64A0, 0RG64A1, 0RG64AJ, 0RG64J0, 0RG64J1, 0RG64JJ, 0RG64K0, 0RG64K1, 0RG64KJ, 0RG64Z0, 0RG64Z1, 0RG64ZJ, 0RG7070, 0RG7071, 0RG707J, 0RG70A0, 0RG70A1, 0RG70AJ, 0RG70J0, 0RG70J1, 0RG70JJ,**

© 2024 Feinstein JA et al. *JAMA Network Open*.

			0RG70K0, 0RG70K1, 0RG70KJ, 0RG70Z0, 0RG70Z1, 0RG70ZJ, 0RG7370, 0RG7371, 0RG737J, 0RG73A0, 0RG73A1, 0RG73AJ, 0RG73J0, 0RG73J1, 0RG73JJ, 0RG73K0, 0RG73K1, 0RG73KJ, 0RG73Z0, 0RG73Z1, 0RG73ZJ, 0RG7470, 0RG7471, 0RG747J, 0RG74A0, 0RG74A1, 0RG74AJ, 0RG74J0, 0RG74J1, 0RG74JJ, 0RG74K0, 0RG74K1, 0RG74KJ, 0RG74Z0, 0RG74Z1, 0RG74ZJ, 0RG8070, 0RG8071, 0RG807J, 0RG80A0, 0RG80A1, 0RG80AJ, 0RG80J0, 0RG80J1, 0RG80JJ, 0RG80K0, 0RG80K1, 0RG80KJ, 0RG80Z0, 0RG80Z1, 0RG80ZJ, 0RG8370, 0RG8371, 0RG837J, 0RG83A0, 0RG83A1, 0RG83AJ, 0RG83J0, 0RG83J1, 0RG83JJ, 0RG83K0, 0RG83K1, 0RG83KJ, 0RG83Z0, 0RG83Z1, 0RG83ZJ, 0RG8470, 0RG8471, 0RG847J, 0RG84A0, 0RG84A1, 0RG84AJ, 0RG84J0, 0RG84J1, 0RG84JJ, 0RG84K0, 0RG84K1, 0RG84KJ, 0RG84Z0, 0RG84Z1, 0RG84ZJ, 0RGA070, 0RGA071, 0RGA07J, 0RGA0A0, 0RGA0A1, 0RGA0AJ, 0RGA0J0, 0RGA0J1, 0RGA0JJ, 0RGA0K0, 0RGA0K1, 0RGA0KJ, 0RGA0Z0, 0RGA0Z1, 0RGA0ZJ, 0RGA370, 0RGA371, 0RGA37J, 0RGA3A0, 0RGA3A1, 0RGA3AJ, 0RGA3J0, 0RGA3J1, 0RGA3JJ, 0RGA3K0, 0RGA3K1, 0RGA3KJ, 0RGA3Z0, 0RGA3Z1, 0RGA3ZJ, 0RGA470, 0RGA471, 0RGA47J, 0RGA4A0, 0RGA4A1, 0RGA4AJ, 0RGA4J0, 0RGA4J1, 0RGA4JJ, 0RGA4K0, 0RGA4K1, 0RGA4KJ, 0RGA4Z0, 0RGA4Z1, 0RGA4ZJ, 0SG0070, 0SG0071, 0SG007J, 0SG00A0, 0SG00A1, 0SG00AJ, 0SG00J0, 0SG00J1, 0SG00JJ, 0SG00K0, 0SG00K1, 0SG00KJ, 0SG00Z0, 0SG00Z1, 0SG00ZJ, 0SG0370, 0SG0371, 0SG037J, 0SG03A0, 0SG03A1, 0SG03AJ, 0SG03J0, 0SG03J1, 0SG03JJ, 0SG03K0, 0SG03K1, 0SG03KJ, 0SG03Z0, 0SG03Z1, 0SG03ZJ, 0SG0470, 0SG0471, 0SG047J, 0SG04A0, 0SG04A1, 0SG04AJ, 0SG04J0, 0SG04J1, 0SG04JJ, 0SG04K0, 0SG04K1, 0SG04KJ, 0SG04Z0, 0SG04Z1, 0SG04ZJ, 0SG1070, 0SG1071, 0SG107J, 0SG10A0, 0SG10A1, 0SG10AJ, 0SG10J0, 0SG10J1, 0SG10JJ, 0SG10K0, 0SG10K1, 0SG10KJ, 0SG10Z0, 0SG10Z1, 0SG10ZJ, 0SG1370, 0SG1371, 0SG137J, 0SG13A0, 0SG13A1, 0SG13AJ, 0SG13J0, 0SG13J1, 0SG13JJ, 0SG13K0, 0SG13K1, 0SG13KJ, 0SG13Z0, 0SG13Z1, 0SG13ZJ, 0SG1470, 0SG1471, 0SG147J, 0SG14A0, 0SG14A1, 0SG14AJ, 0SG14J0, 0SG14J1, 0SG14JJ, 0SG14K0, 0SG14K1, 0SG14KJ, 0SG14Z0, 0SG14Z1, 0SG14ZJ, 0SG3070, 0SG3071, 0SG307J, 0SG30A0, 0SG30A1, 0SG30AJ, 0SG30J0, 0SG30J1, 0SG30JJ, 0SG30K0, 0SG30K1, 0SG30KJ, 0SG30Z0, 0SG30Z1, 0SG30ZJ, 0SG3370, 0SG3371, 0SG337J, 0SG33A0, 0SG33A1, 0SG33AJ, 0SG33J0, 0SG33J1, 0SG33JJ, 0SG33K0, 0SG33K1, 0SG33KJ, 0SG33Z0, 0SG33Z1, 0SG33ZJ, 0SG3470, 0SG3471, 0SG347J, 0SG34A0, 0SG34A1, 0SG34AJ, 0SG34J0, 0SG34J1, 0SG34JJ, 0SG34K0, 0SG34K1, 0SG34KJ, 0SG34Z0, 0SG34Z1, 0SG34ZJ

© 2024 Feinstein JA et al. *JAMA Network Open*.

**Transplant**	N/A	996.8, V42.0, V42.1, V42.2, V42.6, V42.7, V42.81, V42.82, V42.83, V42.84, V43.2<br>**00.91, 00.92, 00.93, 33.5, 33.6, 37.51, 41.0, 41.94, 46.97, 50.5, 52.80, 52.82, 52.83, 52.84, 52.85, 52.86, 55.6**	T86.0, T86.1, T86.2, T86.3, T86.4, T86.5, T86.81, T86.85, T86.89, T86.9, Z94.0, Z94.1, Z94.2, Z94.3, Z94.4, Z94.81, Z94.82, Z94.83, Z94.84<br>**02YA0Z0, 02YA0Z1, 02YA0Z2, 07YP0Z0, 07YP0Z1, 07YP0Z2, 0BYC0Z0, 0BYC0Z1, 0BYC0Z2, 0BYD0Z0, 0BYD0Z1, 0BYD0Z2, 0BYF0Z0, 0BYF0Z1, 0BYF0Z2, 0BYG0Z0, 0BYG0Z1, 0BYG0Z2, 0BYH0Z0, 0BYH0Z1, 0BYH0Z2, 0BYJ0Z0, 0BYJ0Z1, 0BYJ0Z2, 0BYK0Z0, 0BYK0Z1, 0BYK0Z2, 0BYL0Z0, 0BYL0Z1, 0BYL0Z2, 0BYM0Z0, 0BYM0Z1, 0BYM0Z2, 0DY80Z0, 0DY80Z1, 0DY80Z2, 0DYE0Z0, 0DYE0Z1, 0DYE0Z2, 0FY00Z0, 0FY00Z1, 0FY00Z2, 0FYG0Z0, 0FYG0Z1, 0FYG0Z2, 0TY00Z0, 0TY00Z1, 0TY00Z2, 0TY10Z0, 0TY10Z1, 0TY10Z2, 30230AZ, 30230G0, 30230G1, 30230X0, 30230X1, 30230Y0, 30230Y1, 30233AZ, 30233G0, 30233G1, 30230G2, 30230G3, 30230G4, 30230U2, 30230U3, 30230U4, 30230X0, 30230X1, 30230X2, 30230X3, 30230X4, 30230Y0, 30230Y1, 30230Y2, 30230Y3, 30230Y4, 30233AZ, 30233G0, 30233G1, 30233G2, 30233G3, 30233G4, 30233U2, 30233U3, 30233U4, 30233X0, 30233X1, 30233X2, 30233X3, 30233X4, 30233Y0, 30233Y1, 30233Y2, 30233Y3, 30233Y4, 30240AZ, 30240G0, 30240G1, 30240G2, 30240G3, 30240G4, 30240U2, 30240U3, 30240U4, 30240X0, 30240X1, 30240X2, 30240X3, 30240X4, 30240Y0, 30240Y1, 30240Y2, 30240Y3, 30240Y4, 30243AZ, 30243G0, 30243G1, 30243G2, 30243G3, 30243G4, 30243U2, 30243U3, 30243U4, 30243X0, 30243X1, 30243X2, 30243X3, 30243X4, 30243Y0, 30243Y1, 30243Y2, 30243Y3, 30243Y4, 30250G0, 30250G1, 30250X0, 30250X1, 30250Y0, 30250Y1, 30253G0, 30253G1, 30253X0, 30253X1, 30253Y0, 30253Y1, 30260G0, 30260G1, 30260X0, 30260X1, 30260Y0, 30260Y1, 30263G0, 30263G1, 30263X0, 30263X1, 30263Y0, 30263Y1, 3E030U0, 3E030U1, 3E033U0, 3E033U1, 3E0J3U0, 3E0J3U1, 3E0J7U0, 3E0J7U1, 3E0J8U0, 3E0J8U1**

© 2024 Feinstein JA et al. *JAMA Network Open*.

eTable 4. Demographics of Patients With Hospitalizations in the Pediatric Health Information System and MarketScan Medicaid Databases (2009-2019)

Inpatient Hospitalizations 2009-2019			
		PHIS (N=7,186,019)	MarketScan Medicaid (N=2,999,420)
		N (%)	N (%)
**Sex**	Male	3900868 (54.3)	1568574 (52.3)
	Female	3283539 (45.7)	1430783 (47.7)
**Race & Ethnicity**	Non-Hispanic White	3523313 (49)	1298048 (43.3)
	Non-Hispanic Black	1368514 (19)	856193 (28.5)
	Hispanic	1396362 (19.4)	183694 (6.1)
	Other	897830 (12.5)	661485 (22.1)
**Age (years)**	<1	1831621 (25.5)	1434016 (47.8)
	1-4	1851033 (25.8)	425461 (14.2)
	5-9	1275509 (17.7)	239513 (8)
	10-14	1260485 (17.5)	313526 (10.5)
	15-18	967371 (13.5)	586904 (19.6)

© 2024 Feinstein JA et al. *JAMA Network Open*.

eTable 5. Percentage of Patients With CCCs Classified by CCC V2 vs CCC V3 in the Medicaid MarketScan Database (2009-2019)

**MarketScan Medicaid Inpatient Hospitalizations 2009-2019 (N =2,999,420)**			
	**CCC V2**	**CCC V3**	
	Column A	*Column B*	
		CCC Dx, Pr, and Tech Codes	Percentage Change[a,b]
**CCC Category**	N (%)	N (%)	%
Neuromuscular	150558 (5)	158969 (5.3)	5.59
CVD	173781 (5.8)	171876 (5.7)	-1.10
Respiratory	72738 (2.4)	68430 (2.3)	-5.92
Renal	62358 (2.1)	61554 (2.1)	-1.29
GI	118885 (4)	105323 (3.5)	-11.41
Hematologic /Immunologic	122224 (4.1)	116916 (3.9)	-4.34
Metabolic	94609 (3.2)	93256 (3.1)	-1.43
Congenital /Genetic	94441 (3.1)	83139 (2.8)	-11.97
Malignancy	74754 (2.5)	74947 (2.5)	0.26
Neonatal	109191 (3.6)	93930 (3.1)	-13.98
Transplant	12543 (0.4)	17531 (0.6)	39.77
Any CCC	758110 (25.3)	718100 (23.9)	-5.28
Tech Dep	138024 (4.6)	114067 (3.8)	-17.36

[a] (Column D – Column A / Column A) x 100
[b] McNemar's test was used to compare differences in percentages between V2 and V3 by CCC category, which were all significant at p<0.001.

© 2024 Feinstein JA et al. *JAMA Network Open*.

eTable 6. AIC Fit Statistics for Length of Stay and In-Hospital Mortality Regression Models by CCC V2 vs CCC V3 Using Pediatric Health Information System Data (2009-2019)

**Model AIC**				
**Year**	**Length of Stay**		**Mortality**	
	CCC V2	CCC V3	CCC V2	CCC V3
2009	1584262	1588286	46265	46224
2010	1564030	1567528	44605	44272
2011	1593292	1596075	43156	42846
2012	1686763	1688993	43698	43356
2013	1773032	1775576	45209	44978
2014	1823165	1825097	46289	45912
2015	1866780	1868594	46306	46095
2016	1866121	1870645	46828	46929
2017	1876596	1881200	46000	46098
2018	1881433	1885669	44344	44342
2019	1957379	1962191	46097	46149

© 2024 Feinstein JA et al. *JAMA Network Open*.

eFigure 1. The Effects of CCC Version 3 *ICD-10* Code Additions, Deletions, and the Reconceptualization of Technology Codes on the Assignment of CCC Status for Hospitalizations in the Pediatric Health Information System (2019)

Panel 1 of this figure illustrates how codes that were deleted from the V3 system affected the percentages of hospitalizations with and without CCC status. For example, among 717,910 total hospitalizations, 8,024 (1.1%) had a deleted code. After applying the CCC V3 system, 6,217 had a positive CCC status identified by other CCC code(s) and 1,807 had a negative CCC status. Thus, the deleted codes resulted in 1,807 (0.3%) fewer of the total hospitalizations having positive CCC status. Panel 2 of this figure illustrates how codes that were added to the V3 system affected the percentages of hospitalizations with CCC status. For example, among 717,910 total hospitalizations, 16,206 (2.3%) had an added code. After applying the CCC V3 system, 12,137 had a positive CCC status identified by other CCC code(s) and 4,069 had a positive CCC status identified based only on the added code(s). Thus, the added codes resulted in 4,069 (0.6%) more of the total hospitalizations having positive CCC status. Panel 3 of this figure illustrates how reconceptualization of technology codes in the V3 system affected the percentages of hospitalizations with and without CCC status. For example, among 717,910 total hospitalizations, 103,898 (14.5%) had a technology code. After applying the CCC V3 system, 91,110 had a positive CCC status identified by other CCC code(s) and 12,879 had a negative CCC status. Thus, the reconceptualization of the technology codes resulted in 12,879 (1.8%) fewer of the total hospitalizations having positive CCC status. The net effect of each of these changes explains the slightly lower overall percentage of patients identified with positive CCC status by V3 compared to V2.

© 2024 Feinstein JA et al. *JAMA Network Open*.

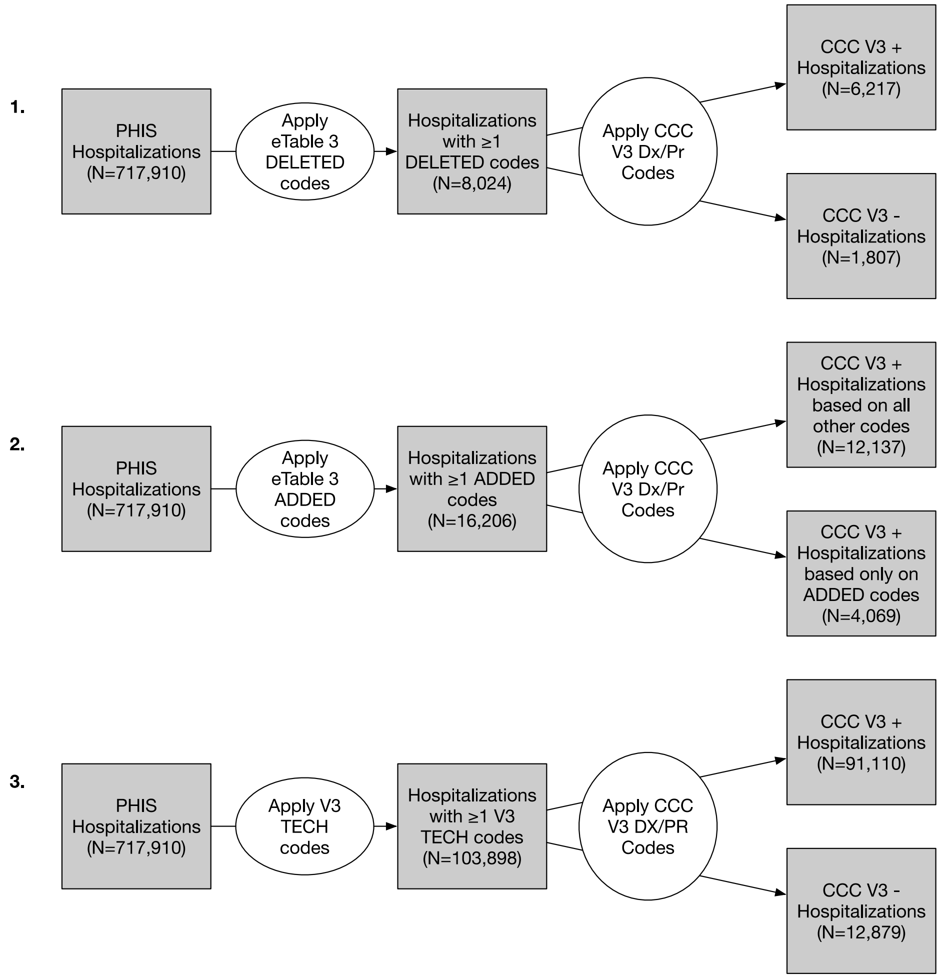

© 2024 Feinstein JA et al. *JAMA Network Open*.

eFigure 2. Percentage of Patients With CCCs in the MarketScan Medicaid Database (2009-2019)

This figure displays the percentages of patients with any, 1, 2, 3, or 4+ CCCs as classified by either CCC V2 or V3 by year. McNemar's test was used to compare differences in percentages between "Any CCC" V2 and V3 by year, which were all significant at p<0.001.

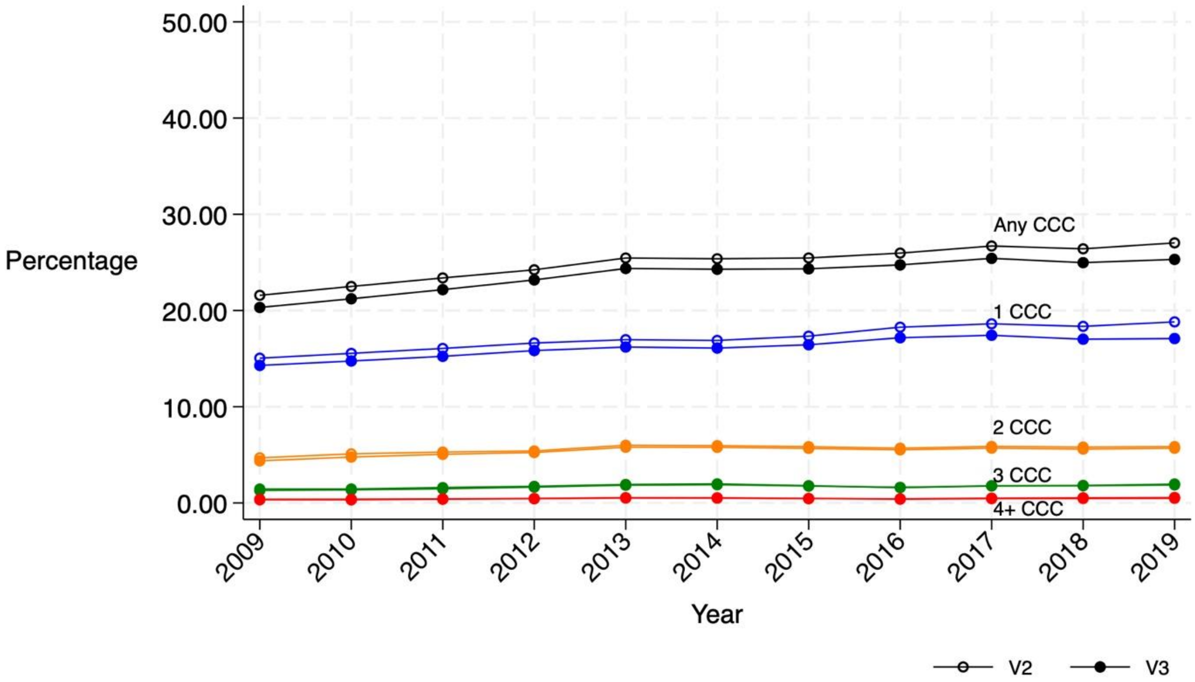

© 2024 Feinstein JA et al. *JAMA Network Open*.

eFigure 3. Comparison of the CCC Systems V2 and V3 in the MarketScan Medicaid Database (2009-2019)

The upper panel of this figure displays the variance in hospital length of stay explained by the presence of a CCC as classified by either CCC V2 or V3 by year. The lower panel of this figure displays the variance in in-hospital mortality explained by the presence of a CCC as classified by either CCC V2 or V3 by year.

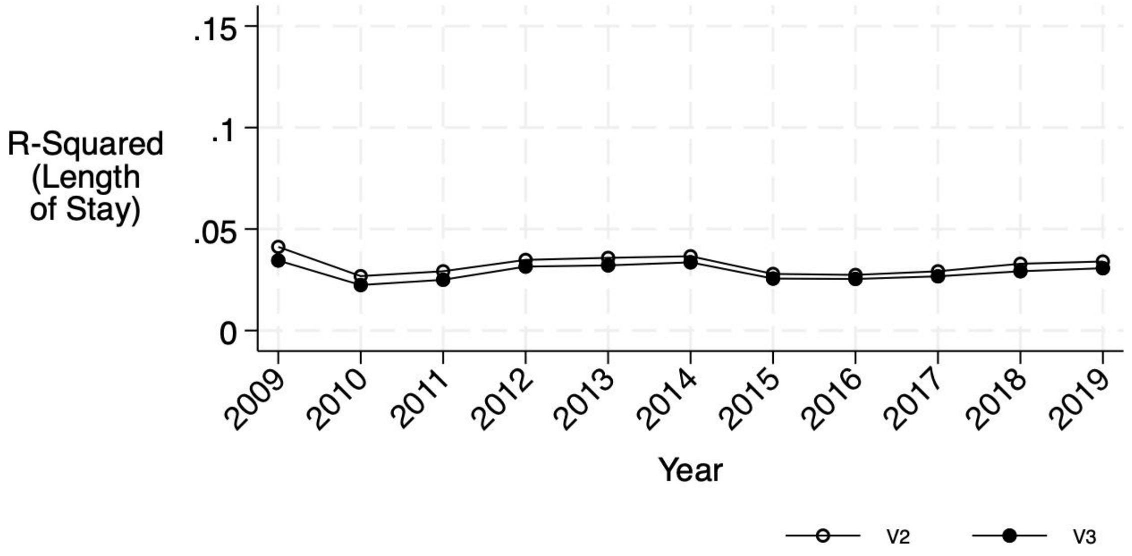

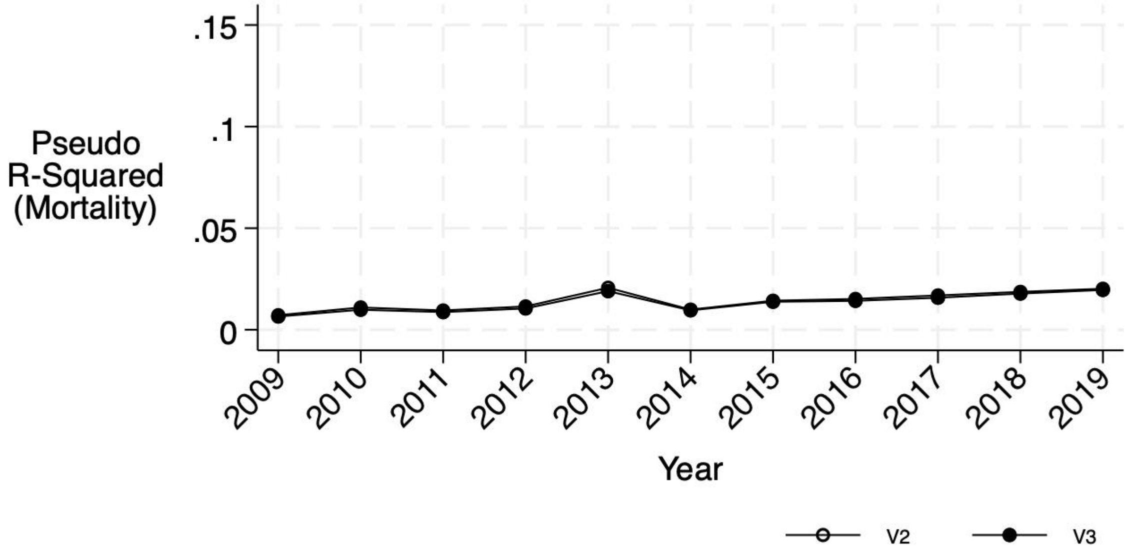

© 2024 Feinstein JA et al. *JAMA Network Open*.
