# Supplementary material for: Pediatric Complex Chronic Condition System Version 3
Source: JAMA Netw Open. 2024 Jul 15;7(7):e2420579. doi: 10.1001/jamanetworkopen.2024.20579 (PMC11250371; doi:10.1001/jamanetworkopen.2024.20579)
Supplement: Supplement 4. — Data Sharing Statement [file jamanetwopen-e2420579-s004.pdf]

Data Sharing Statement

Feinstein. Pediatric Complex Chronic Condition System Version 3. *JAMA Netw Open*.
Published July 15, 2024. doi:10.1001/jamanetworkopen.2024.20579

Data
Data available: Yes
Data types: Data dictionary
How to access data: https://www.childrenshospitals.org/content/analytics/toolkit/complex-chronic-conditions
When available: With publication

Supporting Documents
Document types: None

Additional Information
Who can access the data: Anyone requesting the data.
Types of analyses: For any purpose.
Mechanisms of data availability: Without investigator support.
Any additional restrictions: None.
